# Knowledge evolution and emerging emphasis on outcome standardization in diabetes remission research: a multidatabase bibliometric analysis, 1950 to January 2026

**DOI:** 10.3389/fendo.2026.1943422

**Published:** 2026-10-13

**Authors:** Haotian Qi, Jinying Chen, Han Yang, Ran Bi, Shengxian Xu, Kuisong Wang, Zhenru Liu, Weichen Nie, Xiaoxia Kang, Siwen Cheng, Xinlu Zhang, Rui Chen

**Affiliations:** 1 Changchun University of Chinese Medicine, Changchun, China; 2 The Affiliated Hospital of Changchun University of Chinese Medicine, Changchun, China; 3 The Third Affiliated Clinical Hospital of Changchun University of Chinese Medicine, Changchun, China; 4 College of Basic Medical Sciences, Changchun University of Chinese Medicine, Changchun, China

**Keywords:** bibliometrics, clinical translation, diabetes remission, knowledge evolution, research trends

## Abstract

**Background:**

Diabetes remission has evolved from an infrequently reported clinical observation into an increasingly defined and assessable outcome. Previous bibliometric studies have largely relied on the Web of Science and focused on literature published after 2000, limiting characterization of earlier knowledge foundations and long-term thematic change. This study mapped the global landscape and knowledge evolution of diabetes remission research and examined whether prespecified temporal boundaries were supported by annual publication trends.

**Methods:**

The Web of Science Core Collection, Scopus, and PubMed were searched from database inception to January 30, 2026. After screening, deduplication, and metadata harmonization, 1,554 original articles were included. VOSviewer, CiteSpace, Bibliometrix, and SCImago Graphica were used to analyze publication trends, collaboration networks, journals, authors, keywords, thematic evolution, co-citation clusters, and citation bursts. Segmented Poisson regression was applied to annual publication counts from 1953 to 2025, with prespecified boundaries at 2000 and 2010 and BIC-based breakpoint selection.

**Results:**

Among 1,543 articles published through 2025, the descriptive periods 1950–2000, 2001–2010, and 2011–2025 contained 188, 157, and 1,198 articles, respectively. The prespecified segmented model outperformed the no-breakpoint model (likelihood-ratio test, P < 0.001), with annual percentage changes of 5.19%, 13.00%, and 10.12%, respectively. The slope change after 2000 was significant (P < 0.001), whereas the additional change after 2010 was not (P = 0.115). The BIC-selected model identified one breakpoint at 2001.7 (95% CI, 1996.1–2007.4). The United States, China, and the United Kingdom were the leading contributors. Research themes evolved from case reports, animal studies, and transplantation toward metabolic surgery, weight loss, and lifestyle intervention, with increasing attention to remission definitions, durability, and long-term follow-up.

**Conclusions:**

Diabetes remission research evolved from documentation and foundational work through mechanistic development and evidence integration toward broader clinical evidence and increasing outcome standardization. Publication trends supported a major change in growth around 2000, whereas the 2011 boundary remained descriptive and was supported primarily by thematic rather than statistical evidence.

## 1 Introduction

Diabetes is a major chronic metabolic disease worldwide. Recent estimates from the International Diabetes Federation indicate that the number of adults living with diabetes is likely to continue increasing, with substantial long-term implications for healthcare systems and societies (1). Chronic hyperglycemia is closely associated with microvascular and macrovascular complications, and people with diabetes have an increased risk of cardiovascular disease (2). Conventional diabetes management has primarily focused on sustained glycemic control and reduction of complication risk. However, residual cardiovascular and kidney risk may persist in some patients despite apparently adequate glycemic control (3, 4). With advances in metabolic surgery, intensive weight management, and glucose-lowering therapies that also promote weight loss, some patients may maintain glycemia within the non-diabetic range after withdrawal of glucose-lowering medication. Therapeutic goals have therefore gradually expanded from long-term control toward achieving and maintaining remission.

In 2021, an international expert consensus proposed glycated hemoglobin (HbA1c) below 6.5%, measured at least 3 months after discontinuation of glucose-lowering medication, as the principal criterion for remission of type 2 diabetes (5). This definition helped move remission from an inconsistently interpreted clinical description toward an outcome that can be compared more consistently across studies. Diabetes remission research is also heterogeneous in disease type and disciplinary background. Research on type 2 diabetes remission has largely focused on weight loss and related interventions, including metabolic surgery, energy restriction, and structured weight-management programs, as well as potential mechanisms involving reductions in ectopic fat and recovery of beta-cell function (5–10). By contrast, remission-related research in type 1 diabetes has more often addressed the honeymoon phase or partial clinical remission, immunological interventions, and islet or cell-replacement strategies (11–19). As this body of literature has expanded, narrative reviews alone have become less able to systematically characterize the structural relationships among countries, institutions, authors, research themes, and knowledge foundations (20–24).

Bibliometric methods can characterize a field through publication output, collaboration structures, keywords, and co-citation networks, while also providing information on temporal change. In 2023, Yang et al. analyzed 970 English-language articles published between 2000 and April 2023 in the Web of Science. They reported that the United States, Newcastle University, Roy Taylor, and Obesity Surgery ranked highly at the country, institutional, author, and journal levels, respectively, and that the research emphasis appeared to shift from transplantation and other basic studies toward metabolic surgery and lifestyle intervention (20). In 2025, Liu et al. analyzed 2,254 publications on type 2 diabetes remission in the Web of Science from 2002 to 2022, including 1,903 articles and 351 reviews. They found that the United States, China, and the United Kingdom were the leading contributors; the University of Copenhagen and Lee ranked highly among institutions and authors; bariatric surgery, weight loss, and remission were core keywords; and glucagon-like peptide-1 receptor agonists, low-energy diets, and weight reduction continued to receive attention (21).

These studies established an important foundation for mapping diabetes remission research, but several areas remained open for further investigation. First, both studies relied on a single Web of Science dataset and may therefore have omitted some clinical and interdisciplinary publications indexed in PubMed or Scopus. Second, both analyses began after 2000, limiting their ability to show how earlier case reports, transplantation studies, and immunological interventions contributed to the subsequent knowledge base. Third, previous studies mainly addressed who conducted the research, what topics were studied, and which themes were receiving recent attention. The temporal correspondence among publication trends, keyword evolution, citation bursts, and the co-citation knowledge structure received less detailed consideration.

Accordingly, the present study does not claim to be the first bibliometric analysis of diabetes remission. Instead, it extends existing static knowledge maps by integrating the Web of Science Core Collection, Scopus, and PubMed; expanding the observation period to 1950-January 2026; and interpreting publication trends, trend topics, the thematic map, citation bursts, and co-citation clusters along a shared temporal axis. The study addressed four questions: How is global research activity in diabetes remission distributed? How have research themes and knowledge foundations changed? Do the proposed descriptive periods show an interpretable progression? What additional insights does the present analysis offer relative to the studies published in 2023 and 2025?

## 2 Materials and methods

### 2.1 Data sources and search strategy

The Web of Science Core Collection (WoSCC), Scopus, and PubMed were searched from database inception to January 30, 2026. Search terms covered diabetes mellitus and its major types and synonyms, together with remission-related terms, including remission, reversal, and reverse. Owing to differences in database indexing and search syntax, a topic search was used in WoSCC, whereas Scopus and PubMed searches primarily used title fields. This approach favored broader retrieval in WoSCC and greater specificity in Scopus and PubMed; consequently, retrieval sensitivity was not identical across databases. Complete search strategies are provided in **Supplementary Material 1**.

Document type was restricted to articles in WoSCC and Scopus. In PubMed, original research publication types were eligible, whereas reviews, editorials, and letters were excluded. No language restriction was applied.

### 2.2 Literature screening and data cleaning

The searches identified 4,722 records: 1,700 from WoSCC, 1,587 from Scopus, and 1,435 from PubMed. After document-type and relevance screening, 2,328 records remained; removal of 774 duplicates yielded 1,554 eligible articles. Two reviewers independently screened records, with disagreements resolved through discussion with a third reviewer. Studies addressing the occurrence, intervention, mechanism, prediction, definition, or long-term outcomes of diabetes remission were included. The screening process is shown in **Figure 1**.

**Figure 1:**
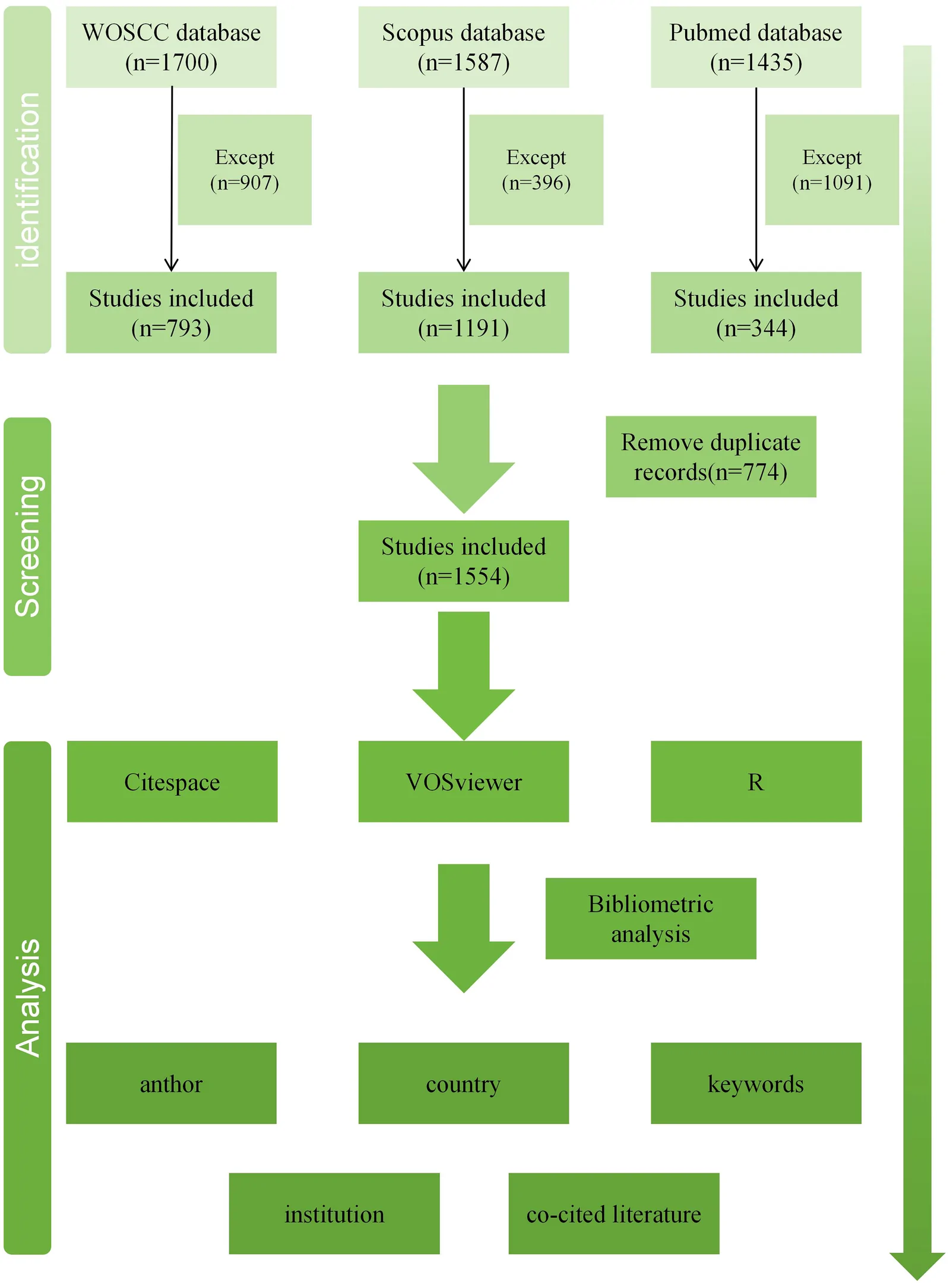
Flow diagram of literature retrieval, screening, and analysis. WoSCC, Scopus, and PubMed yielded 4,722 records. After document-type and relevance screening, 2,328 records remained. Removal of 774 duplicate records resulted in a final dataset of 1,554 articles.

Duplicates were identified primarily by DOI or PMID and, when identifiers were unavailable, by normalized title matching. Discrepant metadata were cross-checked and harmonized, with complementary bibliographic information retained where appropriate. Author initials, country and institution names, keywords, and spelling variants were standardized before analysis.

Country counts were based on all author affiliations, with each country counted once per article; internationally coauthored articles could therefore contribute to more than one country. Institutional name variants referring to the same institution were harmonized, and each standardized institution was counted once per article. Organizationally distinct institutions were retained separately.

Citation-based metrics were derived from WoSCC and Scopus data available at export. Because PubMed did not provide sufficiently complete cited-reference data, co-citation and citation-burst analyses were based on 1,233 eligible WoSCC and Scopus records with adequate reference information. Cited references were analyzed independently of the eligibility criteria applied to the 1,554 source articles and could therefore include original studies, reviews, guidelines, and consensus statements.

### 2.3 Bibliometric analysis

VOSviewer version 1.6.20 was used to analyze author collaboration, journal relationships, author-keyword co-occurrence, and co-cited references (22). Thresholds were five publications for author collaboration, 100 citations for journal co-citation, 10 occurrences for author keywords, and 50 citations for co-cited references. Author keywords were harmonized before analysis.

CiteSpace version 6.4.R1 was used for country collaboration, keyword co-occurrence and clustering, keyword timelines, co-citation clustering, and citation-burst detection (23). The time slice was set to 1 year, and the top five items were retained for each slice. Cluster labels were generated using the log-likelihood ratio algorithm. Citation bursts were identified using the Kleinberg algorithm, with a minimum duration of 2 years.

Bibliometrix version 4.3.0 in R version 4.3.3 was used for database merging, journal ranking, Bradford’s law, cumulative journal trends, trend-topic analysis, and thematic mapping (24).

SCImago Graphica was used to create the geographical map of country-level publication activity.

### 2.4 Temporal stratification and interpretive framework

Publication output was summarized in 5-year intervals. Changes in keywords, thematic structure, citation bursts, and co-citation patterns were considered jointly to define descriptive periods of research development. These periods were used as an interpretive framework rather than as statistically determined change points. Supporting co-citation patterns and citation-burst references are summarized in **Supplementary Table 5**.

Annual publication counts from 1953 to 2025 were further examined using segmented Poisson regression with a log link; 2026 was excluded because the search covered only part of the year. Fixed boundaries at 2000 and 2010, derived from the descriptive periodization, were evaluated using hinge terms, and segment-specific annual percentage changes were calculated as \(100 \times [\exp(\beta)-1]\).

A separate data-driven analysis evaluated models with 0–3 estimated breakpoints using the segmented() procedure, with BIC used for model selection and at least five annual observations required per segment. Bootstrap restarting used 100 restarts (seed = 2026), and the breakpoint 95% confidence interval was estimated using the delta method. Overdispersion was assessed using the Pearson dispersion statistic, with quasi-Poisson regression used as a sensitivity analysis. Residual temporal dependence was examined using the Pearson-residual ACF and Ljung–Box tests at lags 5, 10, and 15. A two-sided P < 0.05 was considered statistically significant. The segmented regression and related diagnostic analyses were performed in R version 4.5.2 using the segmented package (version 2.2.1).

### 2.5 Ethics statement

This study used publicly available bibliometric information and did not involve identifiable patient data, clinical interventions, or biological samples. Ethics approval was therefore not required.

## 3 Results

### 3.1 Literature screening and publication trends

After screening and deduplication, 1,554 articles were included. **Figure 1** summarizes literature retrieval, screening, deduplication, and analysis. Publication counts for the 5-year intervals from 1950–1955 through 1996–2000 were 2, 6, 11, 3, 7, 15, 23, 41, 43, and 37, respectively. A total of 188 articles were published between 1950 and 2000, accounting for 12.1% of the dataset; this period was classified as the early accumulation period within the descriptive temporal framework.

There were 64 publications in 2001–2005 and 93 in 2006–2010, for a combined total of 157 articles (10.1%). Although the absolute publication volume remained lower than after 2011, the annual growth rate had already increased; this interval was therefore classified as a transitional growth period within the descriptive temporal framework.

Publication counts increased to 250 in 2011–2015, 390 in 2016–2020, and 558 in 2021–2025. The 1,198 articles published during 2011–2025 accounted for 77.1% of the dataset and indicated a marked expansion in publication volume. Annual output reached 127 articles in 2025, the highest count among complete calendar years. Eleven articles were identified for 2026 through January 30; because this represented an incomplete year, it was not compared directly with previous full-year counts. Publication output by 5-year interval and the descriptive temporal framework are shown in **Figure 2** and **Supplementary Table 1**.

**Figure 2:**
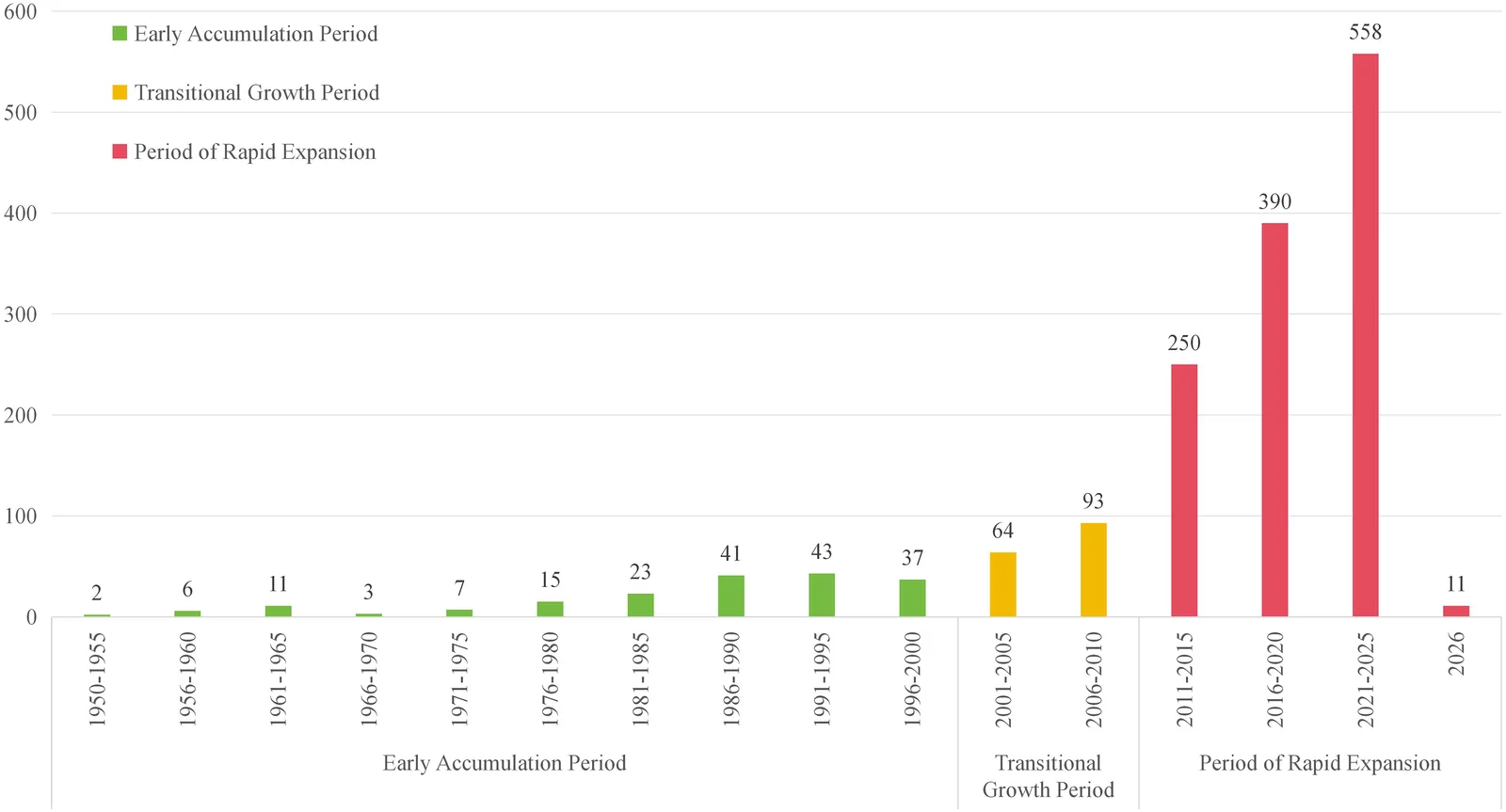
Publication output in 5-year intervals and descriptive periods of diabetes remission research. Green, yellow, and red indicate the early accumulation period, transitional growth period, and period of rapid expansion in publication volume, respectively. The three periods were used to interpret thematic evolution and were not all defined by statistical breakpoints; segmented Poisson regression results are presented in **Table 1**. Data for 2026 include publications indexed only through January 30.

The prespecified fixed-boundary model provided a better fit than the no-breakpoint model (AIC, 384.066 vs 423.058; BIC, 393.228 vs 427.638; likelihood-ratio P < 0.001). The estimated APCs were 5.19% (95% CI, 4.06%–6.34%) during 1953–2000, 13.00% (95% CI, 10.33%–15.73%) during 2001–2010, and 10.12% (95% CI, 8.83%–11.42%) during 2011–2025 (all P < 0.001). The slope change after 2000 was statistically significant (β = 0.0716, 95% CI, 0.0399–0.1032; P < 0.001), whereas the additional slope change after 2010 was not (β = −0.0258, 95% CI, −0.0578 to 0.0063; P = 0.115) (**Table 1**).

**Table 1:** Segmented Poisson regression analysis of annual publication trends in diabetes remission research, 1953-2025.

A. Model comparison				
Model	AIC	BIC	Likelihood-ratio test P value	
No-breakpoint Poisson model	423.058	427.638	—	
Prespecified fixed-boundary model (2000 and 2010)	384.066	393.228	<0.001	
BIC-selected data-driven breakpoint model	386.506	395.668	—	

B. Annual percentage change by prespecified period				
Period	APC, %	95% CI	P value	
1953-2000	5.19	4.06-6.34	<0.001	
2001-2010	13.00	10.33-15.73	<0.001	
2011-2025	10.12	8.83-11.42	<0.001	

C. Boundary tests and data-driven breakpoint				
Analysis	Parameter	Estimate	95% CI	P value
Primary Poisson model	Change in log slope after 2000	0.0716	0.0399 to 0.1032	<0.001
Primary Poisson model	Change in log slope after 2010	−0.0258	−0.0578 to 0.0063	0.115
Data-driven breakpoint analysis	Breakpoint year	2001.7	1996.1 to 2007.4	—
Quasi-Poisson sensitivity	Change in log slope after 2000	0.0716	0.0330 to 0.1101	<0.001
Quasi-Poisson sensitivity	Change in log slope after 2010	−0.0258	−0.0648 to 0.0132	0.192

The likelihood-ratio test compares the prespecified fixed-boundary model with the no-breakpoint Poisson model. “BIC-selected data-driven breakpoint model” denotes the model selected by BIC only within the separate candidate set containing 0–3 freely estimated breakpoints; the prespecified fixed-boundary model was not included in that BIC-selection procedure. The data-driven analysis selected one breakpoint at 2001.7 (95% CI, 1996.1–2007.4).

The data-driven breakpoint confidence interval was calculated using the delta method. The Pearson dispersion statistic for the prespecified Poisson model was 1.429. The quasi-Poisson sensitivity analysis used the same fixed-boundary specification and did not change the interpretation of the boundary tests. Residual temporal dependence was examined using the Pearson-residual autocorrelation function and Ljung–Box tests; P values at lags 5, 10, and 15 were 0.208, 0.321, and 0.238, respectively, providing no evidence of material residual temporal dependence. The segmented regression included 1,543 publications from 1953–2025; 11 publications from incomplete year 2026 were excluded.

Within the separate data-driven candidate set, BIC selected a one-breakpoint model with an estimated breakpoint at 2001.7 (95% CI, 1996.1–2007.4). The prespecified fixed-boundary model had a lower BIC than the data-driven model (393.228 vs 395.668); however, the prespecified model was not included in the data-driven BIC-selection procedure. Both analyses were consistent with a major change in publication growth around 2000.

The Pearson dispersion statistic was 1.429. In the quasi-Poisson sensitivity analysis, the slope change after 2000 remained statistically significant (β = 0.0716, 95% CI, 0.0330–0.1101; P < 0.001), whereas the change after 2010 remained nonsignificant (β = −0.0258, 95% CI, −0.0648 to 0.0132; P = 0.192), consistent with the primary analysis. Examination of the Pearson-residual ACF showed no systematic residual autocorrelation pattern, and Ljung–Box tests at lags 5, 10, and 15 were nonsignificant (P = 0.208, 0.321, and 0.238, respectively). Thus, the 2011 boundary was retained as a descriptive boundary reflecting changes in research emphasis rather than as a statistically supported change point in publication growth.

### 3.2 Distribution of countries, regions, and institutions

Research activity was concentrated mainly in North America, Europe, and East Asia. The United States produced the largest number of articles (n = 365), followed by China (n = 176), the United Kingdom (n = 132), Italy (n = 63), and Canada (n = 61). Because internationally coauthored articles could be attributed to more than one country, country-level publication counts were not interpreted as mutually exclusive proportions of the overall dataset. The United States received 21,080 citations and had a total link strength of 186, ranking first in publication output, citations, and collaboration strength (**Figure 3**).

**Figure 3:**
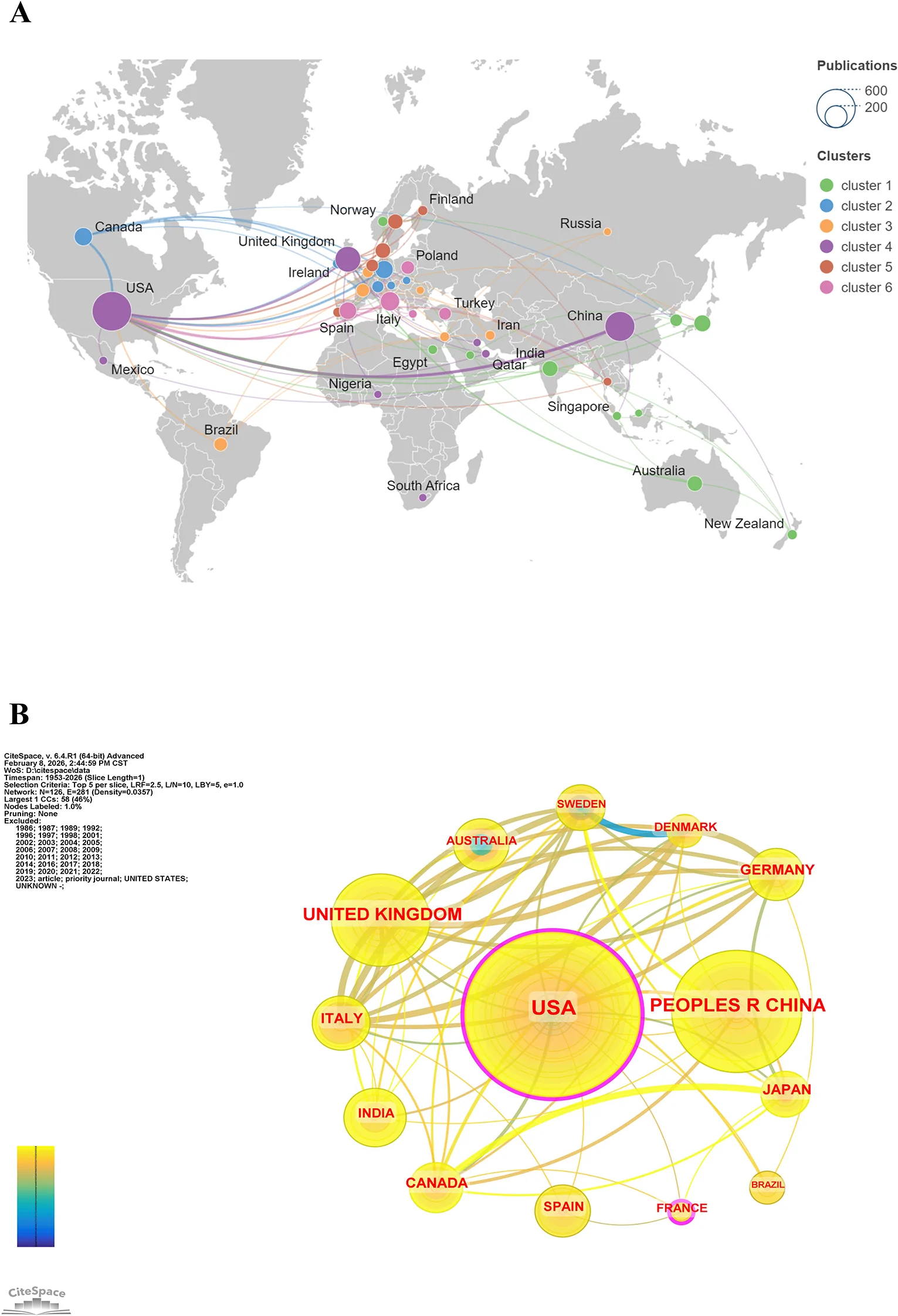
Country and regional distribution and international collaboration network in diabetes remission research. **(A)** Geographical distribution of publications: circle size represents publication output and colors indicate network clusters. **(B)** Country collaboration network: node size represents publication frequency, links represent collaboration, and purple outer rings indicate betweenness centrality.

In the country collaboration network, the United States showed prominent links with China, Italy, Canada, and Germany, whereas the United Kingdom had comparatively strong links with the United States, Germany, Canada, and Switzerland. France and the United States had betweenness centrality values of 0.17 and 0.15, respectively. Although France produced only 26 articles, it had the highest centrality value, suggesting that publication volume and the bridging role between collaboration groups were not fully aligned.

At the institutional level, Newcastle University had the highest publication output (n = 58), followed by the University of Glasgow (n = 46), Harvard University (n = 33), the University of Florida (n = 28), and the University of Washington (n = 22). The 15 most productive institutions included organizations from multiple countries, with institutions from the United Kingdom and the United States particularly well represented. Institutional output is shown in **Figure 4** and **Supplementary Table 3**.

**Figure 4:**
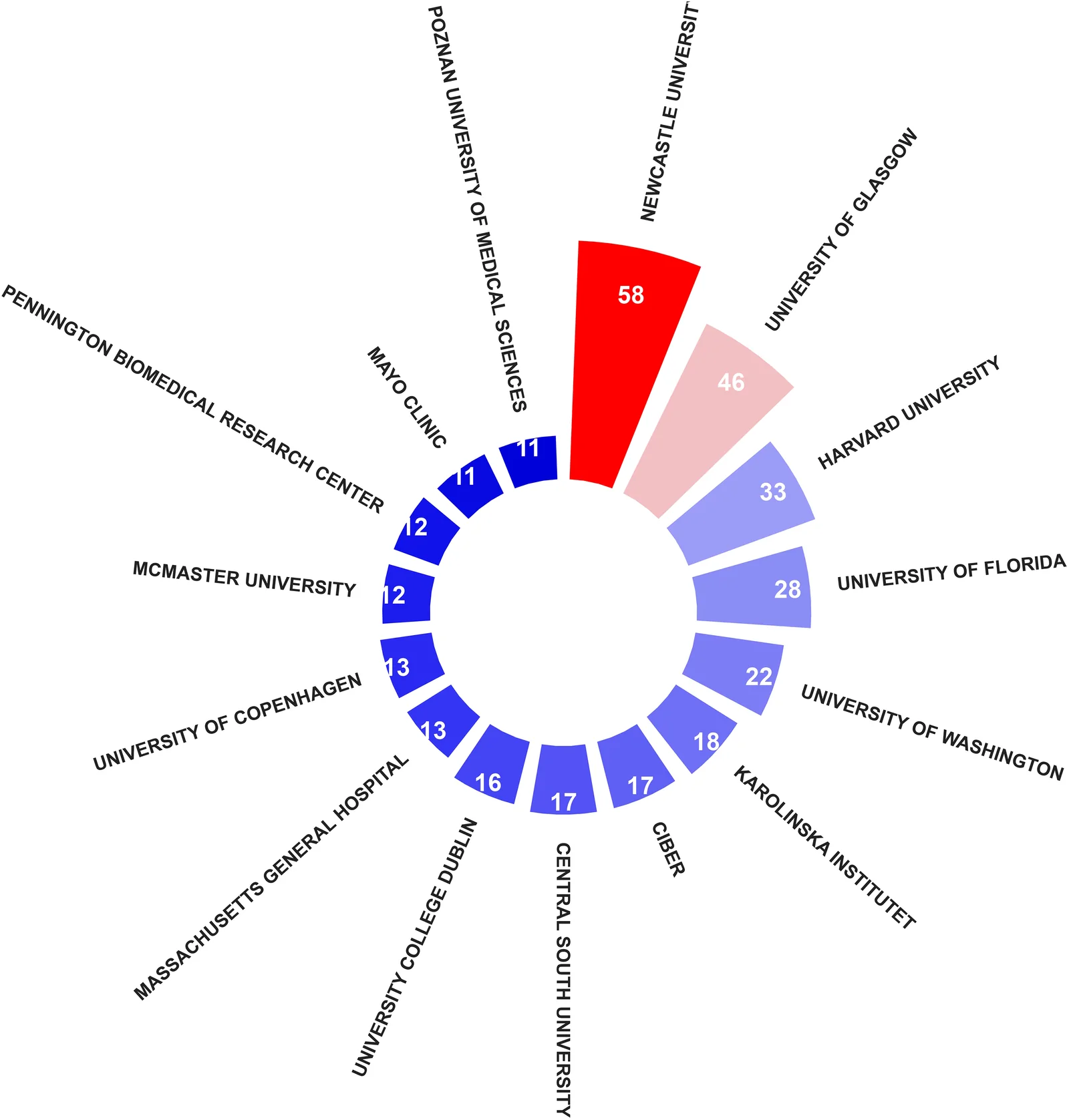
The 15 institutions with the highest publication output. Bar length and numerical labels represent the number of unique publications attributed to each standardized institution. Recognized institutional name variants were harmonized before counting, and each institution was counted at most once per article.

### 3.3 Journal and author profiles

A total of 520 journals published articles on diabetes remission. The five most productive journals were Obesity Surgery (n = 116), Diabetes (n = 71), Diabetes Care (n = 70), Diabetologia (n = 38), and Surgery for Obesity and Related Diseases (n = 30). The 10 most productive journals contributed 436 articles (28.1%), and eight were classified in the 2024 Journal Citation Reports Q1 category. Journal rankings are presented in **Table 2**.

**Table 2:** Top 10 journals publishing the highest number of publications in the field of diabetes remission.

Source	Documents	2024 IF	JCR
Obesity Surgery	116	3.1	Q1
Diabetes	71	7.5	Q1
Diabetes Care	70	16.6	Q1
Diabetologia	38	10.2	Q1
Surgery for Obesity and Related Diseases	30	3.8	Q1
Diabetic Medicine	29	3.4	Q2
Diabetes Obesity & Metabolism	24	5.7	Q1
Diabetes Research and Clinical Practice	20	7.4	Q1
Frontiers in Endocrinology	19	4.6	Q1
PLOS ONE	19	2.6	Q2

Cumulative publication curves for the core journals showed substantially greater absolute increases after 2011, reflecting the higher publication volume during this period. Bradford’s law indicated that a relatively small group of journals in diabetology, obesity research, and metabolic surgery constituted the core source set. The dual-map overlay suggested that the knowledge base drew on clinical medicine, molecular biology, immunology, genetics, and surgery. Diabetes Care, Diabetes, and Obesity Surgery occupied relatively central positions in the journal relationship and co-citation networks. These results are shown in **Figures 5A–F**.

**Figure 5:**
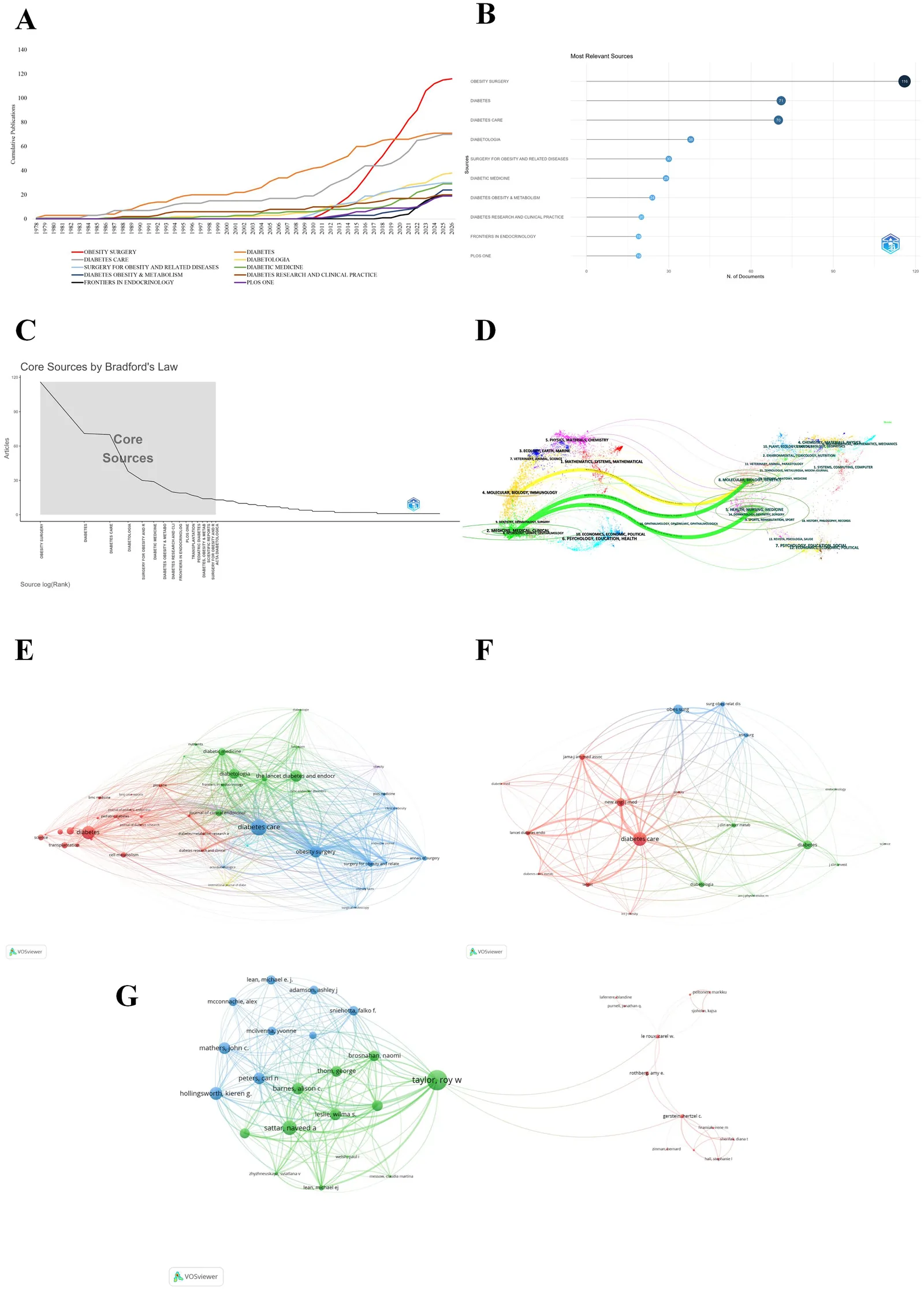
Journal structure and Author collaboration network in diabetes remission research. **(A)** Cumulative publication trends for 10 core journals. **(B)** The 10 journals with the highest publication output. **(C)** Distribution of core journals according to Bradford’s law. **(D)** Dual-map overlay of journals: curves indicate citation pathways across disciplines. **(E)** Journal relationship network. **(F)** Co-cited journal network. **(G)** Author collaboration network: Nodes represent authors, links represent collaborative relationships, and colors indicate different collaboration groups.

Roy Taylor published 37 articles, received 5,320 citations, and had a total link strength of 214. Naveed A. Sattar published 24 articles, received 3,682 citations, and had a total link strength of 176. Michael E. J. Lean published 22 articles, and Wilma S. Leslie published 16. Alison C. Barnes, Louise McCombie, and George Thom were also among the most productive authors. The author collaboration network showed discernible clustering, with close links among researchers associated with Newcastle University and the University of Glasgow, while connections between some groups were comparatively limited. **Figure 5G** presents the author network, and **Table 3** provides detailed metrics.

**Table 3:** Top 10 authors with the highest number of publications in the field of diabetes remission.

Author	Documents	Citations	Total link strength	Institution	Country
Taylor, Roy	37	5320	214	Newcastle University	UK
Sattar, Naveed A.	24	3682	176	University of Glasgow	UK
Lean, Michael E.J.	22	1041	133	University of Glasgow	UK
Leslie, Wilma S.	16	3022	156	University of Glasgow	UK
Barnes, Alison C.	15	3258	158	Newcastle University/University of Glasgow	UK
McCombie, Louise	15	2992	151	University of Glasgow	UK
Thom, George	14	2910	145	University of Glasgow	UK
Atkinson, Mark A.	13	940	10	University of Florida	USA
Ghanem, Omar M.	13	133	37	Department of Surgery, Mayo Clinic	USA
Le Roux, Carel W.	13	921	14	Ulster University	UK

### 3.4 Keywords, trend topics, and thematic structure

In the unfiltered CiteSpace keyword dataset, diabetes mellitus, human, male, female, remission, and non insulin dependent diabetes mellitus occurred 906, 812, 713, 659, 617, and 505 times, respectively. Terms such as human, male, female, adult, and controlled study largely reflected database indexing and study-population characteristics and were therefore not interpreted independently as specific scientific hotspots. Keyword frequencies are listed in **Supplementary Table 4**, and the co-occurrence network is shown in **Figure 6**.

**Figure 6:**
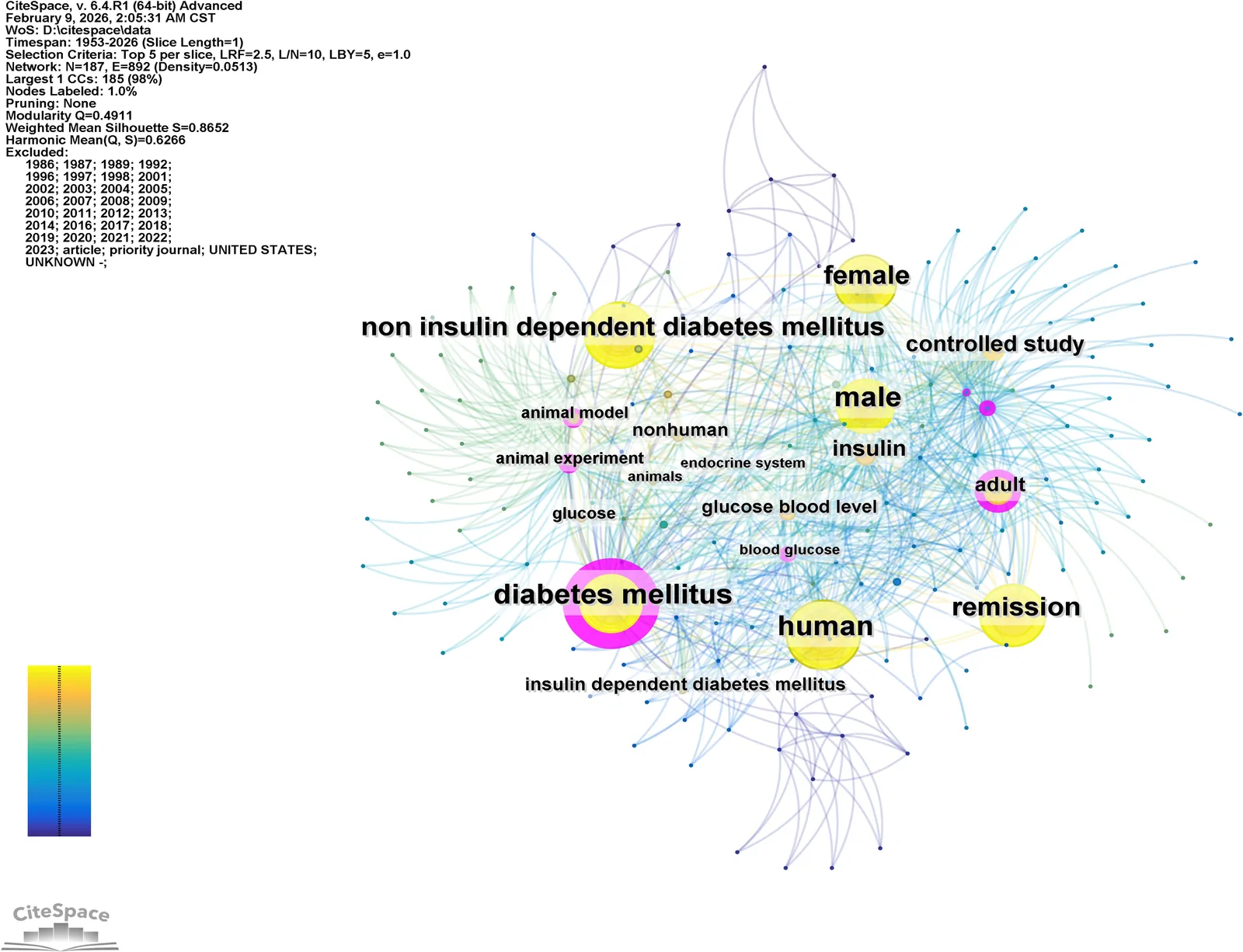
Keyword co-occurrence network. Node size represents keyword frequency, links represent co-occurrence, and colors indicate clusters.

Automated CiteSpace clustering generated labels including diabetic diet, adult, case report, endocrine system, diabetic nephropathy, and head/wounds and injuries. The timeline indicated that several themes persisted over extended periods. Because some cluster labels appeared to be influenced by broad indexing terms, these results were used primarily to illustrate historical coverage and were not considered sufficient on their own to define research frontiers. The keyword clusters and timeline are shown in **Figure 7**.

**Figure 7:**
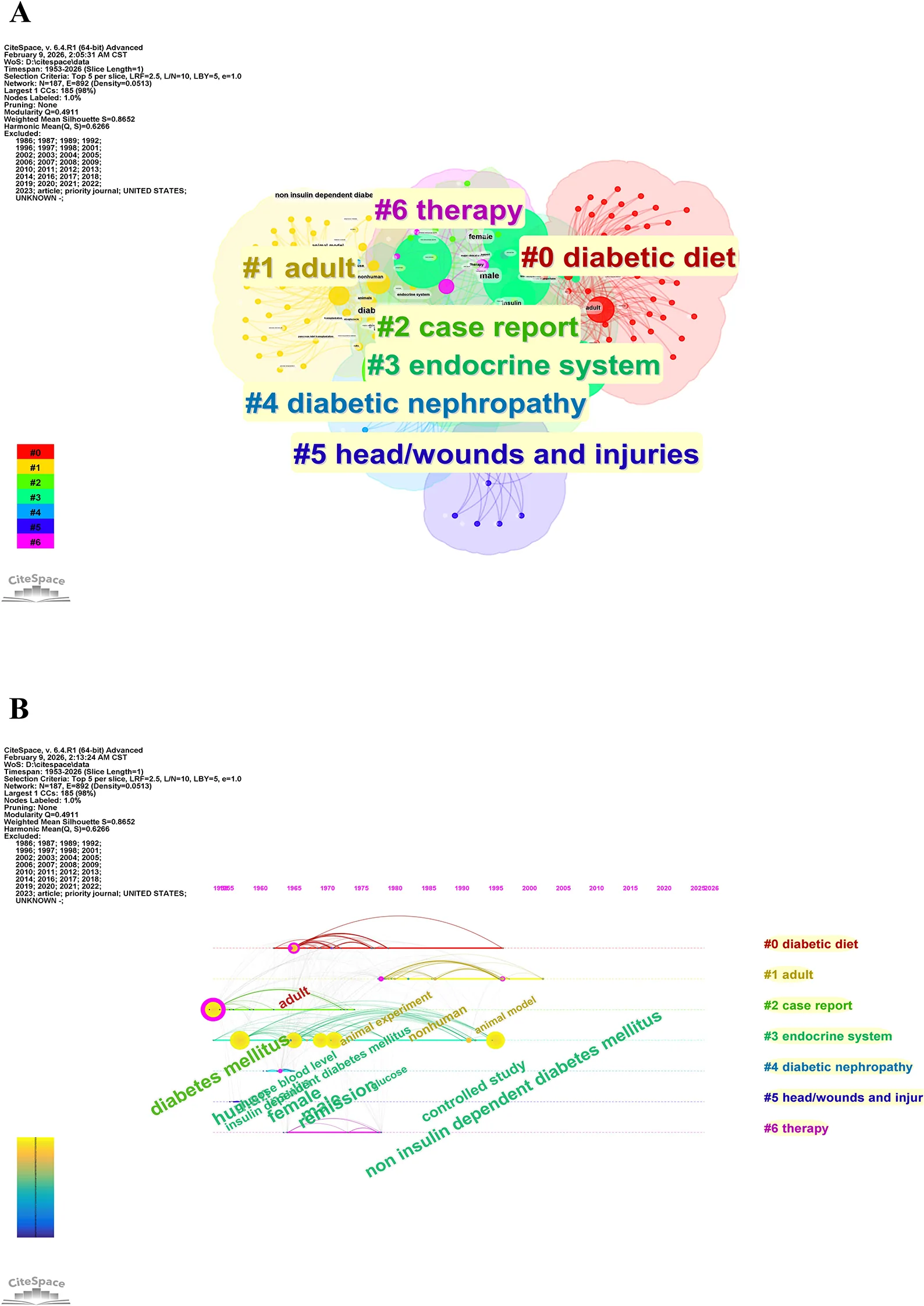
Keyword clusters and timeline. **(A)** CiteSpace keyword cluster map. **(B)** Keyword cluster timeline showing the emergence and persistence of themes.

In the author-keyword network, type 2 diabetes mellitus, bariatric surgery, diabetes remission, weight loss, and lifestyle intervention occupied relatively central positions. The annual keyword heatmap showed that terms related to type 2 diabetes, bariatric surgery, and remission appeared more frequently in recent years. These results are presented in **Figure 8**.

**Figure 8:**
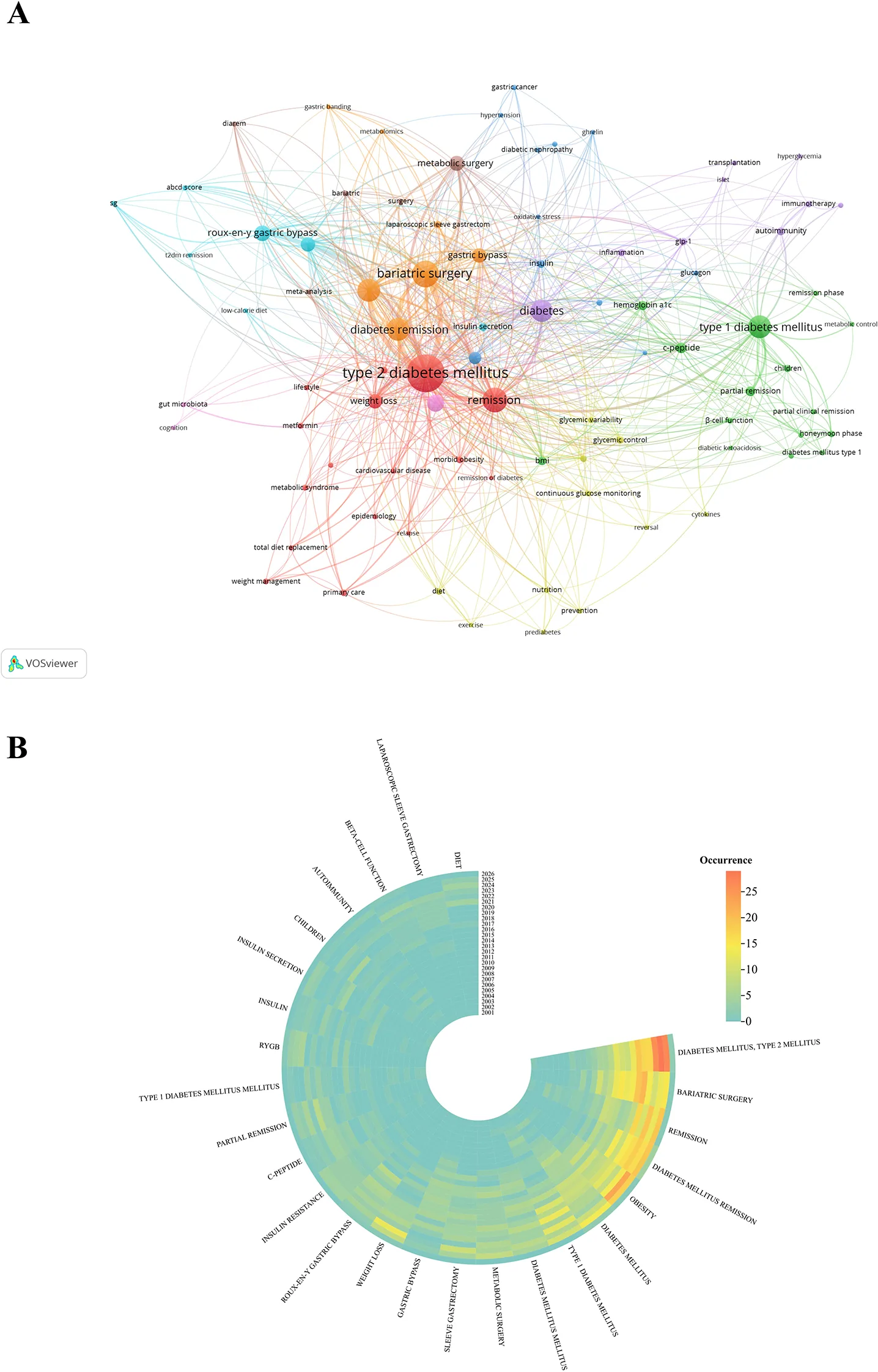
Author keywords and annual variation. **(A)** Author-keyword co-occurrence network. **(B)** Circular heatmap of annual keyword frequency: warmer colors indicate higher occurrence.

Trend-topic analysis showed that earlier studies more often addressed rat, animal experiment, glucose, insulin, pancreas transplantation, child, and adolescent. Later topics included gastric bypass, obesity, weight loss, type 2 diabetes, diabetes remission, exercise, and lifestyle intervention. In the thematic map, type 2 diabetes, remission, bariatric surgery, obesity, and diabetes remission were located in the basic-themes quadrant, indicating broad connections with other topics. Honeymoon phase, clinical remission, and partial clinical remission were located in the motor-themes quadrant, suggesting a relatively developed and active specialty related to partial remission in type 1 diabetes. Trend topics and the thematic map are shown in **Figure 9**.

**Figure 9:**
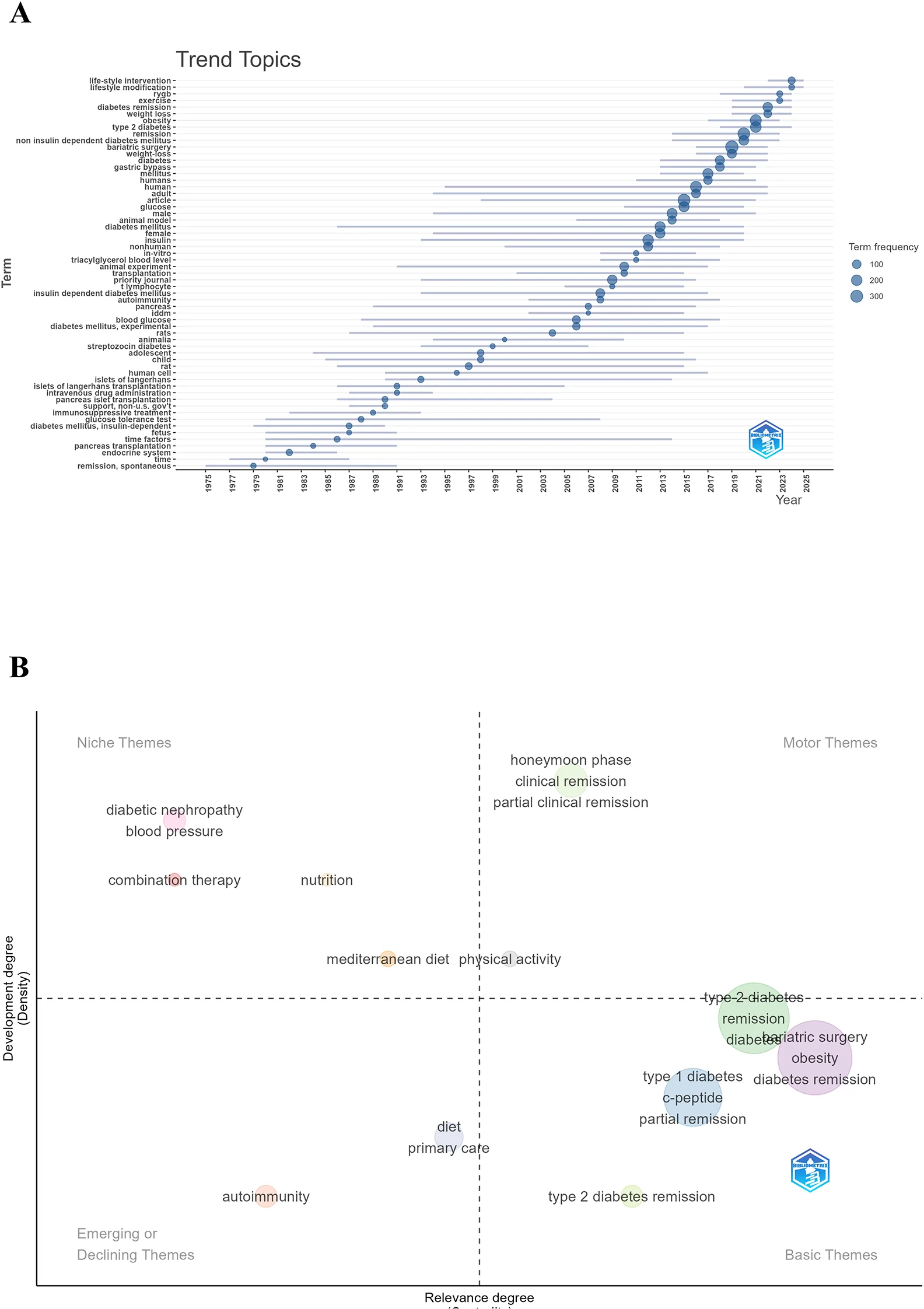
Evolution of research themes. **(A)** Trend-topic map: bubble size represents term frequency and horizontal lines indicate the active period of each topic. **(B)** Thematic map: the horizontal axis represents centrality and the vertical axis represents density. The upper-right, upper-left, lower-left, and lower-right quadrants correspond to motor, niche, emerging or declining, and basic themes, respectively.

### 3.5 Citation bursts and the co-citation knowledge structure

Citation-burst analysis identified the 2008 randomized trial of adjustable gastric banding by Dixon et al. as having the highest burst strength (3.13). The 2007 study by Adams et al. on long-term mortality after gastric bypass had a burst strength of 2.21. These references suggest that the efficacy and longer-term outcomes of metabolic surgery were important components of the knowledge base during the earlier expansion of the field. The 30 references with the strongest citation bursts are shown in **Figure 10**.

**Figure 10:**
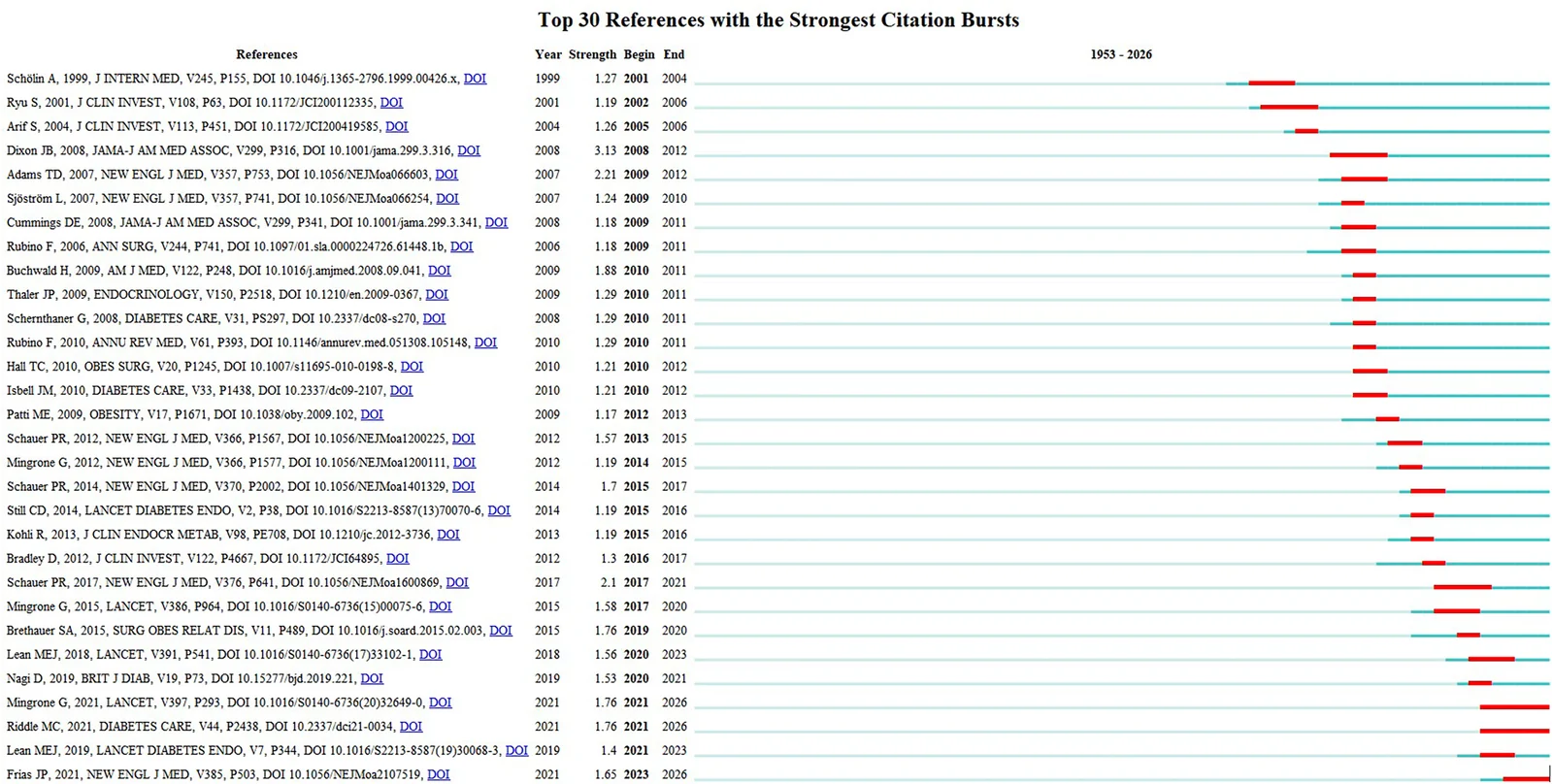
The 30 co-cited references with the strongest citation bursts. Red segments indicate the duration of citation bursts, and Strength represents burst intensity.

Representative references with ongoing or recently initiated citation bursts included the 10-year follow-up of metabolic surgery by Mingrone et al. and the consensus definition of remission by Riddle et al., both with burst strengths of 1.76. The tirzepatide study by Frías et al. had a strength of 1.65, the DiRECT trial 1.56, the UK position statement on diabetes remission 1.53, and the 2-year DiRECT follow-up 1.40. Further details are provided in **Table 4**.

**Table 4:** Representative references showing recent citation bursts in diabetes remission research.

Rank	Title	First author	Journal	DOI	Strength
1	Primary care-led weight management for remission of type 2 diabetes (DiRECT): an open-label, cluster-randomised trial	Michael EJ Lean	Lancet	10.1016/S0140-6736(17)33102-1	1.56
2	Remission of type 2 diabetes: a position statement from the Association of British Clinical Diabetologists (ABCD) and the Primary Care Diabetes Society (PCDS)	Dinesh Nagi	British Journal of Diabetes	10.15277/bjd.2019.221	1.53
3	Metabolic surgery versus conventional medical therapy in patients with type 2 diabetes: 10-year follow-up of an open-label, single-centre, randomised controlled trial	Geltrude Mingrone	Lancet	10.1016/S0140-6736(20)32649-0	1.76
4	Consensus Report: Definition and Interpretation of Remission in Type 2 Diabetes	Matthew C Riddle	Diabetes Care	10.2337/dci21-0034	1.76
5	Durability of a primary care-led weight-management intervention for remission of type 2 diabetes: 2-year results of the DiRECT open-label, cluster-randomised trial	Michael EJ Lean	Lancet Diabetes & Endocrinology	10.1016/S2213-8587(19)30068-3	1.40
6	Tirzepatide versus Semaglutide Once Weekly in Patients with Type 2 Diabetes	Juan P Frías	New England Journal of Medicine	10.1056/NEJMoa2107519	1.65

Co-citation clustering indicated that earlier knowledge bases included cure, mechanism, and mediating remission. Clusters related to metabolic remodeling and physiology became visible in the intermediate period, whereas more recent clusters included defining remission, duration, long-term follow-up, intestinal alteration, and cross-sectional population-based study. Overall, the knowledge structure appeared to progress from establishing whether remission was possible, to explaining potential mechanisms, and then to increasing attention to remission definitions, durability, and population-level evaluation. The co-citation knowledge structure is shown in **Figure 11**. The relationships among the descriptive periods, documented co-citation patterns, representative citation-burst references, burst periods, and their interpretive relevance are summarized in **Supplementary Table 5**.

**Figure 11:**
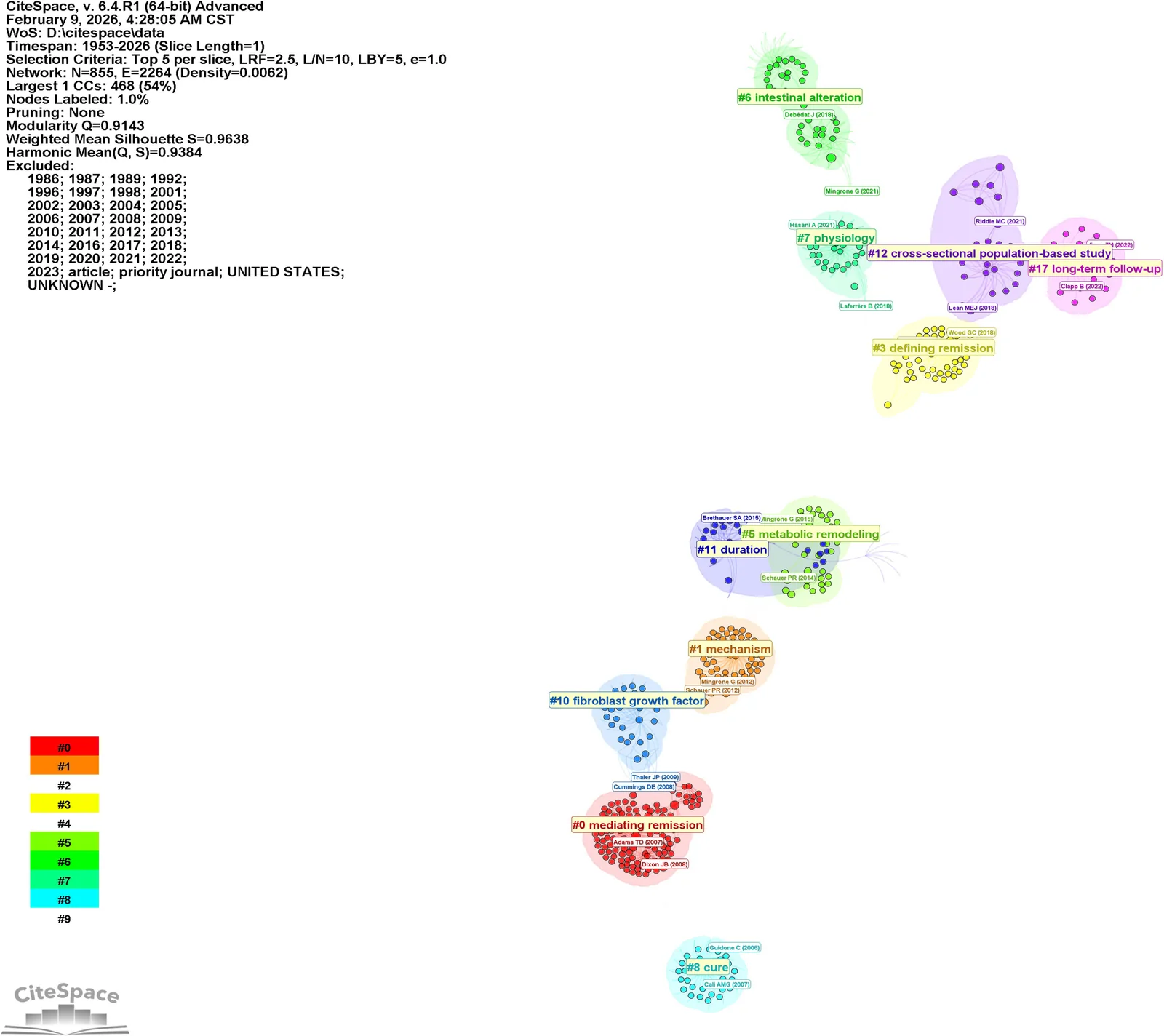
Knowledge structure based on reference co-citation analysis. Colored regions represent knowledge clusters and illustrate a shift from efficacy and mechanism toward remission definitions, duration, and long-term follow-up.

## 4 Discussion

### 4.1 Convergence with and differences from the 2023 and 2025 studies, and additional insights from the present study

The present findings were consistent with several stable conclusions from earlier bibliometric studies. All three analyses identified the United States as a major contributor, Roy Taylor and related research groups as influential participants, Obesity Surgery as a highly productive journal, and metabolic surgery, weight loss, and lifestyle intervention as core themes in diabetes remission research (20, 21). These convergent findings suggest that the main research centers and central topics have remained relatively stable despite differences in databases, time periods, and inclusion criteria.

Differences in sample size and rankings across studies should not necessarily be interpreted as conflicting results. The 2023 study included 970 English-language articles published from 2000 to April 2023 and reported the United States, Newcastle University, Roy Taylor, and Obesity Surgery as leading contributors (20). The 2025 study focused exclusively on type 2 diabetes remission and included 2,254 articles and reviews published from 2002 to 2022; the University of Copenhagen, Lee, and Obesity Surgery ranked highly among institutions, authors, and journals (21). The present study included original research on remission in both type 1 and type 2 diabetes from three databases and extended the observation period back to approximately 1953. Differences in search fields, disease scope, document types, database coverage, and name harmonization would be expected to affect document counts and the rankings of institutions, authors, and journals. Comparisons are therefore more informative when focused on the consistency and evolution of the knowledge structure than on absolute counts alone.

The main addition of the present study was not a repeated identification of metabolic surgery and lifestyle intervention as hotspots, but the joint interpretation of several temporal signals to examine how these themes emerged. Trend-topic analysis indicated that earlier studies more often involved case reports, animal experiments, glucose metabolism, and transplantation, followed by increasing attention to gastric bypass, obesity, weight loss, and lifestyle intervention (**Figure 9A**). Citation bursts extended from randomized trials and long-term outcome studies of metabolic surgery to DiRECT, the remission consensus definition, and long-term follow-up (**Figure 10**, **Table 4**). Co-citation clusters also shifted from cure and mechanism toward defining remission, duration, and long-term follow-up (**Figure 11**). The temporal correspondence between these documented bibliometric patterns and our historical interpretation is summarized in **Supplementary Table 5**. Collectively, these findings are consistent with increasing attention to the definition, durability, and standardization of remission outcomes in the more recent literature; however, this interpretation represents a descriptive synthesis of multiple bibliometric signals rather than evidence of a discrete historical transition at 2011. Taken together, these findings support a gradual movement from documenting remission and exploring mechanisms toward defining, maintaining, and comparing remission as a clinical outcome.

The inclusion of remission-related studies in type 1 diabetes also allowed honeymoon phase, clinical remission, and partial clinical remission to be positioned within the thematic map (**Figure 9B**). This branch was not represented in the 2025 analysis, which focused on type 2 diabetes. Multidatabase integration broadened the coverage of clinical and interdisciplinary literature, although it also introduced challenges related to non-equivalent search fields and incomplete comparability of citation data. Overall, the additional value of the present study lies in its broader historical range, multidatabase coverage, and use of several temporal signals to interpret a progression from documentation of remission and mechanistic development toward expansion of clinical evidence and increasing attention to outcome standardization. A detailed comparison of the three studies is provided in **Supplementary Table 6**.

Annual publication trends provided statistical support for an acceleration around 2000. The data-driven segmented model estimated the main breakpoint at 2001.7, with a 95% CI that included the prespecified 2000 boundary; this timing was broadly consistent with the increasing visibility of mechanistic studies and remission-assessment themes. In contrast, although the APC declined from 13.00% in 2001–2010 to 10.12% in 2011-2025, the change in slope after 2010 was not statistically significant. The year 2011 should therefore not be interpreted as an independent statistical breakpoint confirmed by publication counts. It was retained as a descriptive boundary because long-term follow-up, remission definitions, and outcome assessment became more prominent in the citation-burst and co-citation structures during this period. Thus, the three periods are more appropriately viewed as an interpretive framework for knowledge evolution than as a phase structure determined entirely by publication trends.

### 4.2 Period I (1950-2000): documentation of remission and accumulation of pathophysiological evidence

A total of 188 articles were published between 1950 and 2000, representing 12.1% of the included literature. Although this period spanned more than half a century, average annual output was low and publication counts varied across 5-year intervals. Case report, animal model, glucose, insulin, and pancreas transplantation were prominent in the keyword timeline and trend-topic analysis, suggesting that early research mainly documented remission phenomena and developed preliminary mechanistic hypotheses.

In 1953, Cheng reported spontaneous remission in a patient with marked hyperglycemia and ketosis (25), and subsequent reports described remission in specific endocrine or clinical contexts (26). These observations suggested that hyperglycemia might not be irreversible under all circumstances. However, the small number of cases, evolving diagnostic frameworks, and heterogeneous outcomes—including normoglycemia, insulin withdrawal, or symptom resolution—limited the development of a coherent evidence base. The separate case report cluster was consistent with the fragmented observational character of this period.

From the 1970s onward, research began to move from passive observation toward experimental intervention. Studies of glycemic improvement after weight-reduction surgery, transplantation of fetal pancreas or pancreatic islets, allogeneic islet transplantation, and immunosuppressive therapy examined potential reversibility through reduced metabolic burden, replacement of functional beta cells, or suppression of autoimmune injury (11–15, 27). Near-normoglycemic remission and recovery of beta-cell function in newly diagnosed type 2 diabetes also received increasing attention (28–30). These topics aligned with early co-citation clusters labeled cure and mechanism, indicating that the literature was initially concerned with whether diabetes could be reversed and which biological processes might be involved.

The relatively slow publication growth during this period may partly reflect the limited level of evidence and the restricted generalizability of available interventions. Case reports, small observational series, and animal experiments were unlikely to displace clinical management centered on long-term glycemic control and prevention of complications. The DCCT and UKPDS further reinforced the importance of glycemic control for reducing complications (31, 32). In addition, early weight-reduction procedures were associated with substantial complications, transplantation was constrained by donor availability and immune rejection, and immunosuppressive therapy raised safety concerns. Remission was therefore more often regarded as a phenomenon occurring under particular conditions than as a broadly attainable clinical outcome.

Increasing standardization of etiologic diabetes classification around 1998 (33) provided a conceptual basis for the later divergence of remission research in type 1 and type 2 diabetes. Type 2 diabetes studies increasingly focused on body weight, insulin resistance, and beta-cell function, whereas type 1 diabetes studies more often addressed immune injury and residual beta-cell function. The main contribution of the first period may therefore be viewed as establishing the existence of remission and defining the initial questions that guided several later research pathways.

### 4.3 Period II (2001-2010): mechanistic development and integration of multiple evidence pathways

There were 64 publications in 2001–2005 and 93 in 2006-2010, representing a 45.3% increase between the two intervals. Segmented regression further showed that the APC during 2001–2010 was 13.00%, compared with 5.19% during 1953-2000, and that the slope change after 2000 was statistically significant. This period should therefore not be characterized as one of slow publication growth. Rather, it represented a marked acceleration in growth, although the absolute publication volume remained lower than after 2011. Animal model, nonhuman, insulin, and controlled study remained visible in the trend topics, suggesting that basic experiments and small-scale clinical studies continued to play an important role. At the same time, co-citation clusters related to mechanism and metabolic remodeling became more prominent, indicating a shift from description toward mechanistic interpretation.

In type 2 diabetes, the central questions gradually shifted from whether surgery was effective to why it was effective and which patients might benefit. Rubino et al. reported that the glycemic effects of gastrointestinal bypass were not fully dependent on weight loss, stimulating research on the proximal intestine and gut-pancreas signaling (6). Taylor’s twin-cycle hypothesis interpreted the reversibility of type 2 diabetes in relation to ectopic fat accumulation in the liver and pancreas (7). Pournaras et al. subsequently described early improvements in insulin resistance and secretory function after surgery (34), whereas Hall et al. identified diabetes duration, preoperative control, and treatment characteristics as factors associated with postoperative remission (35). These developments suggest movement from average treatment effects toward mechanistic explanation and patient stratification.

In type 1 diabetes, interventions targeting CD3, CD20, and low-dose interleukin-2 showed potential to preserve or restore beta-cell function in animal models (16–18). The insulin dose-adjusted HbA1c definition proposed in 2009 provided a more standardized quantitative measure of partial remission in children and adolescents (19). The position of honeymoon phase, clinical remission, and partial clinical remission in the motor-themes quadrant also suggested that partial remission in type 1 diabetes had developed into a relatively coherent specialty.

Large-scale clinical expansion did not yet parallel the mechanistic advances of this period. Long-term follow-up, direct comparisons between surgery and intensive medical treatment, standardized definitions of remission, and cost-effectiveness evidence remained limited. Studies used different criteria, including fasting glucose, HbA1c, insulin discontinuation, or continued use of selected medications, which constrained direct comparisons. Immunological interventions in type 1 diabetes also faced challenges related to translation from animal models, limited durability, and safety.

The principal contribution of 2001–2010 was therefore not the establishment of mature clinical programs, but the development of multiple lines of investigation involving metabolic-surgery mechanisms, ectopic fat, immunomodulation, patient selection, and remission assessment. The marked acceleration in publication growth around 2000, together with the emergence of mechanism and metabolic remodeling clusters, suggests that this period linked early observation with the later expansion of clinical evidence.

### 4.4 Period III (2011-2025): expansion of clinical evidence and increasing attention to outcome standardization

Between 2011 and 2025, 1,198 articles were published, accounting for 77.1% of the dataset, and absolute output increased across three consecutive 5-year intervals. The APC remained significantly positive at 10.12%, but the slope change after 2010 was not statistically significant. This period was therefore defined mainly by publication scale and thematic change rather than by an independently supported statistical breakpoint. **Figure 5A** shows that cumulative output from core journals rose more sharply after 2011. In the cumulative journal networks for the full observation period, Obesity Surgery had the highest publication output, while Diabetes, Diabetes Care, and Diabetologia also occupied productive or central positions. The dual-map overlay further suggested connections with surgery, clinical medicine, molecular biology, and immunology.

Cumulative author and institutional results indicated that leading UK-based groups were influential in the field. Roy Taylor, Naveed A. Sattar, Michael E. J. Lean, Wilma S. Leslie, and their collaborators formed a closely connected network, while the University of Glasgow and Newcastle University ranked highly among institutions. Considered alongside representative studies published after 2011, the low-energy diet and ectopic-fat research led by Taylor’s group and the primary care weight-management studies led by the Glasgow group provided a sequence of mechanistic and clinical evidence for type 2 diabetes remission. Lim et al. reported in 2011 that severe energy restriction was accompanied by reductions in liver and pancreatic fat and recovery of beta-cell function (8). The DiRECT trial later showed that structured weight management delivered in primary care could induce remission in a proportion of participants and that remission was closely associated with maintenance of weight loss (9, 10).

Randomized trials and long-term follow-up of metabolic surgery further strengthened its clinical evidence base. The 5-year STAMPEDE results, the 10-year follow-up by Mingrone et al., and an earlier randomized trial reported higher long-term remission proportions after metabolic surgery than after conventional medical treatment in selected patients (36–38). The SOS study suggested that long-term remission after surgery may also be associated with improved microvascular and macrovascular outcomes (39). Prediction tools such as DiaRem and studies in Asian patients with lower body mass index extended the discussion toward individualized patient selection (40, 41).

In the cumulative country collaboration network, the United States ranked first in publication output, citations, and total link strength, indicating broad participation in knowledge production and collaboration. China ranked second in publication output but had lower betweenness centrality and total link strength than the United States and the United Kingdom, suggesting that growth in research volume had not translated to the same extent into a bridging role in international collaboration. France had a comparatively small publication volume but the highest centrality, indicating that some countries may exert network influence primarily by connecting otherwise separate research groups. These findings describe cumulative structures across the observation period and do not imply that all differences in collaboration emerged after 2011.

A notable feature of the descriptive 2011–2025 period was not only the expansion of intervention pathways, but also increasing attention to remission assessment, long-term follow-up, and comparisons across therapeutic approaches. The consensus report by Riddle et al., the long-term follow-up by Mingrone et al., the DiRECT studies, the UK position statement, and the tirzepatide trial all showed recent citation bursts (5, 9, 10, 37, 42, 43). Clusters labeled defining remission, duration, and long-term follow-up were consistent with these burst references and suggested that research questions had increasingly shifted from whether remission could occur to how it should be defined, how long it could be maintained, and when relapse might occur.

New incretin-based therapies have broadened clinical options for weight management. Tirzepatide has been associated with substantial reductions in HbA1c and body weight (42), but favorable glycemic control during ongoing therapy should not automatically be equated with remission after medication withdrawal. Under the 2021 international consensus, remission should be assessed at least 3 months after discontinuation of glucose-lowering medication (5). Future pharmacological studies would benefit from distinguishing metabolic control during treatment, remission after withdrawal, and the subsequent duration of remission.

### 4.5 From recognition of remission to clinical outcome management: research logic and future directions

The three descriptive periods are best viewed not as isolated historical segments, but as a continuum in which the main research questions gradually evolved. The first period focused primarily on documenting whether diabetes remission could occur and on proposing possible biological explanations through case reports and experimental studies. The second period placed greater emphasis on the mechanisms underlying remission, together with factors relevant to patient selection and outcome assessment. During the third period, randomized trials, long-term follow-up studies, and international consensus statements contributed to the development of remission as a clinical outcome that could be defined, compared, and monitored over time. Because segmented regression provided statistical support only for the first boundary around 2000, the three-period framework was used primarily to interpret thematic evolution rather than to define three statistically distinct stages of development.

Across these periods, the evidence base broadened from case reports, small observational studies, and animal experiments to include mechanistic investigations, randomized controlled trials, long-term follow-up studies, and consensus reports. Intervention-related research also expanded from transplantation, immunosuppression, and relatively high-risk surgical procedures to metabolic surgery with a growing evidence base, structured weight-management programs, and newer glucose-lowering therapies associated with weight reduction. At the thematic level, the co-citation structure showed a shift from cure and mechanism toward metabolic remodeling, followed by increasing attention to defining remission, duration, and long-term follow-up. Taken together, these patterns support an interpretation of the field’s development as a progression from documentation of remission and accumulation of foundational evidence, through mechanistic investigation and evidence integration, to expansion of clinical evidence and increasing attention to outcome standardization.

Within this broader trajectory, research related to type 1 and type 2 diabetes appeared to follow related but partly distinct pathways. In the earlier literature, themes commonly associated with type 1 diabetes included pancreatic or islet transplantation, immunological intervention, residual beta-cell function, and partial clinical remission. Within the overall thematic structure, honeymoon phase, clinical remission, and partial clinical remission formed a relatively coherent area of research. By contrast, research related to type 2 diabetes expanded from glycemic improvement after bariatric or metabolic surgery to mechanisms of metabolic surgery, energy restriction, structured weight management, reduction of ectopic fat, and recovery of beta-cell function. More recent publications have increasingly addressed remission definitions, durability, relapse, and long-term outcomes. Trend topics and thematic mapping indicated partly distinct research strands for the two diabetes types, whereas author keywords and recent citation bursts appeared to reflect mainly the expansion of type 2 diabetes remission research. These findings suggest that, although both strands appeared to evolve from clinical observation toward mechanistic investigation and outcome assessment, their thematic trajectories and principal research priorities differed.

The criteria used to assess remission in the two diabetes types are not directly interchangeable because they reflect different clinical and biological contexts. Type 2 diabetes remission generally refers to the maintenance of glycemic measures below the diagnostic threshold for diabetes for a specified period after withdrawal of glucose-lowering medication. Partial remission in type 1 diabetes is more commonly assessed using insulin requirements and HbA1c, with some studies also incorporating measures of residual endogenous insulin secretion. The increasing attention to outcome standardization identified in this analysis may therefore be interpreted as reflecting mainly the development of unified definitions, assessment of remission durability, and long-term follow-up in type 2 diabetes. It also includes a related but relatively independent movement toward more quantitative assessment of partial remission in type 1 diabetes. Because the included publications were not classified individually by diabetes type, these differences should be regarded as an interpretation based on the thematic structure and representative literature rather than as evidence of a statistically tested association between diabetes type and publication year.

Prominent foundational themes in the field included type 2 diabetes, bariatric surgery, obesity, weight loss, and lifestyle intervention. Their positions in the thematic map suggest broad connections with other research topics, but thematic position alone is insufficient to identify a topic as a recent research frontier. When the thematic findings are considered alongside recent citation bursts and co-citation clusters, several priorities for future research emerge.

A first priority is further refinement of remission outcomes and reporting practices according to diabetes type. Studies of type 2 diabetes would benefit from explicitly reporting the HbA1c threshold, duration of medication withdrawal, assessment time points, duration of remission, criteria for relapse, and resumption of glucose-lowering treatment. For type 1 diabetes, clearer distinctions among the honeymoon phase, partial clinical remission, and complete remission may improve comparability, together with more consistent reporting of insulin requirements, HbA1c, and residual beta-cell function.

Greater attention could also be given to the maintenance of remission rather than remission attainment alone. The 2-year follow-up of DiRECT, long-term studies of metabolic surgery, and clusters related to duration and long-term follow-up suggest that weight regain, changes in beta-cell function, behavioral adherence, and treatment discontinuation may contribute to relapse. More systematic evaluation of these factors in long-term studies could help clarify the durability of remission and the conditions under which it is maintained.

Direct comparative studies of different intervention pathways would also be informative. Using prespecified remission criteria, future studies could compare metabolic surgery, structured weight-management programs, and pharmacological weight-loss interventions in terms of effectiveness, safety, durability, cost, and patient acceptability. Combined and sequential strategies may also warrant investigation. In pharmacological studies, it would be useful to distinguish metabolic control during continued treatment from remission after treatment withdrawal and to report the duration of any subsequent remission.

Broader geographic and population representation remains another relevant consideration. Publication activity was concentrated among a relatively small number of highly productive countries, while highly productive institutions and authors were concentrated mainly in the United Kingdom, the United States, and a limited number of European countries. Multicenter research could be expanded in Asia, Africa, Latin America, and low- and middle-income settings. Greater inclusion of individuals with lower BMI, longer diabetes duration, younger age, and diverse ethnic backgrounds may also help assess the generalizability of current evidence.

Finally, greater collaboration among research groups may facilitate integration across intervention pathways and diabetes types. The author collaboration network contained several relatively concentrated groups, with limited connections between some of them. This pattern suggests opportunities for further exchange among researchers working on different treatment approaches and disease-specific remission concepts. The development of separate core outcome sets for type 1 and type 2 diabetes, together with shared minimum reporting elements where appropriate, may improve comparability without treating the two remission constructs as equivalent. Shared long-term follow-up databases and interdisciplinary comparative studies may further support evidence integration.

### 4.6 Strengths and limitations

This study has several strengths. First, the combined use of the Web of Science Core Collection (WoSCC), Scopus, and PubMed broadened the literature coverage and may have reduced potential biases arising from differences in journal indexing, disciplinary scope, and historical coverage across individual databases. Compared with previous studies that relied primarily on WoSCC and largely focused on publications after 2000, the present study covered a longer period. This extended timeframe facilitated examination of the development of diabetes remission research from early case observations and experimental investigations to clinical trials, long-term follow-up studies, and increasing attention to outcome standardization.

Second, following literature screening, deduplication, and harmonization of author and institutional names, this study integrated analyses of annual publication trends, national and institutional collaboration, author networks, journal distribution, keyword evolution, thematic mapping, citation bursts, and co-citation clusters. These approaches captured complementary dimensions of the field, including research volume, collaboration patterns, thematic content, and the underlying knowledge base. Several findings showed broadly consistent patterns across analyses. For example, the increase in annual publication output after 2011 occurred in parallel with the cumulative growth of publications in core journals. The increasing prominence of weight loss and remission among author keywords was consistent with citation bursts involving DiRECT-related publications. Similarly, recent clusters concerning remission definitions, duration, and long-term follow-up corresponded with the increasing influence of international consensus statements and long-term clinical studies. This cross-method consistency may reduce the uncertainty associated with interpreting the evolution of the field solely on the basis of a single keyword network or publication curve.

Third, in addition to the descriptive periodization, segmented Poisson regression was used to examine annual publication trends. The findings supported a principal change point around 2000 but did not indicate a statistically significant change in slope after 2010. This analysis allowed statistically supported changes in publication trends to be distinguished from interpretive boundaries derived from thematic evolution, thereby avoiding characterization of all periods as statistically validated stages. Comparisons among the prespecified-boundary model, the no-breakpoint model, and the BIC-selected breakpoint model also provided a relatively transparent statistical basis for the proposed periodization.

Fourth, this study considered not only high-frequency themes and highly cited publications but also thematic position, temporal emergence, and citation bursts when interpreting emerging research directions. This approach may help distinguish relatively stable foundational themes from topics showing a more recent increase in influence and provides a closer connection between the bibliometric findings and the discussion of remission definitions, remission maintenance, and long-term outcome assessment.

Several limitations should also be acknowledged. First, the database-specific search fields were not identical. The broader topic search in WoSCC may have provided greater retrieval sensitivity, whereas the primarily title-based searches in Scopus and PubMed prioritized specificity and may have missed relevant records whose remission-related terminology appeared only in abstracts or indexing fields. Although the use of three databases broadened overall coverage, this field asymmetry may have influenced the composition of the final corpus. In addition, PubMed records did not provide cited-reference data with the completeness required for reference-level analyses. Therefore, co-citation and citation-burst analyses were based on the 1,233 eligible WoSCC and Scopus source records with sufficiently complete cited-reference data, rather than all 1,554 eligible source articles.

Second, bibliometric indicators are influenced by publication age, database coverage, and citation practices. Earlier publications have had more time to accumulate citations, whereas recently published studies with potential clinical relevance may not yet have achieved high citation counts or detectable citation bursts. Citation counts and burst strength should therefore be interpreted primarily as indicators of knowledge dissemination and academic influence rather than as direct measures of evidence quality or clinical value.

Third, although the study included publications related to remission in both type 1 and type 2 diabetes, the records were not individually classified by diabetes type, and separate thematic networks were not constructed for the two disease types. Comparisons of their research pathways and temporal evolution were therefore based mainly on keywords, thematic mapping, co-citation clusters, and representative publications. These comparisons should be regarded as exploratory interpretations rather than as evidence of a statistically tested association between diabetes type and publication year.

Finally, the three periods were defined primarily according to publication volume and temporal changes in research themes. Segmented Poisson regression supported a principal change point around 2000 but did not support a significant change in slope after 2010; the 2011 boundary therefore remained interpretive. Although the Pearson dispersion statistic of 1.429 did not indicate substantial overdispersion, annual publication counts may still have been influenced by the expansion of database coverage and the overall growth of global research output. In addition, only publications indexed through January 30 were included for 2026, and this incomplete year was not directly comparable with previous full calendar years. Future studies could use relative publication proportions and Joinpoint regression or other change-point methods for external validation and could further construct disease-specific or period-specific thematic networks.

## 5 Conclusion

Building on bibliometric studies published in 2023 and 2025, this study expanded the data sources to WoSCC, Scopus, and PubMed; extended the observation period to 1950-January 2026; and combined publication trends, thematic mapping, citation bursts, co-citation clusters, and segmented regression to interpret the long-term knowledge evolution of diabetes remission research.

The findings suggest that the United States, the United Kingdom, and several core research groups contributed substantially to knowledge production and accumulation of clinical evidence, while research output from China expanded considerably. Metabolic surgery, weight loss, and lifestyle intervention remained stable basic themes, whereas remission definitions, duration, long-term follow-up, and relapse assessment received increasing attention. Segmented regression primarily supported a significant change in publication trends around 2000, whereas the 2011 boundary did not receive independent statistical support.

Compared with previous analyses, the main contribution of this study was not another ranking of productive countries or hotspot keywords, but an account of how current themes developed from early case reports and experimental research and were subsequently reframed as definable, comparable, and longitudinally assessed clinical outcomes. The evolution of diabetes remission research may be summarized as documentation and foundational accumulation, mechanistic development and evidence integration, and expansion of clinical evidence with increasing attention to outcome standardization. These three periods should be regarded as an interpretive framework based on converging thematic evidence.

## Supplementary material

Supplementary Material 1 Complete search strategies for Web of Science, PubMed, and Scopus.

Supplementary Material 2 Supplementary tables for publication trends, countries/regions, institutions, keywords, temporal correspondence among descriptive periods and citation/co-citation patterns, and comparison with bibliometric studies published in 2023 and 2025.
