# Supplementary material for: Knowledge evolution and emerging emphasis on outcome standardization in diabetes remission research: a multidatabase bibliometric analysis, 1950 to January 2026
Source: Front Endocrinol (Lausanne). 2026 Oct 13;17:1943422. doi: 10.3389/fendo.2026.1943422 (PMC13711509; doi:10.3389/fendo.2026.1943422)
Supplement: Supplementary file 1 [file Supplementaryfile1.docx]

**Search Query**

Web of science

TS=((“diabetes remission” or “diabetes mellitus remission” or “type 2 diabetes remission” or “type 2 diabetes mellitus remission” or “T2DM remission” or “remission of diabetes” or “remission of diabetes mellitus” or “remission of type 2 diabetes” or “remission of type 2 diabetes mellitus” or “reversal of diabetes” or “reversal of diabetes mellitus” or “reversal of type 2 diabetes” or “reversal of type 2 diabetes mellitus” or “reversal of T2DM” or “reverse diabetes” or “reverse diabetes mellitus” or “reverse type 2 diabetes” or “ reverse type 2 diabetes mellitus” or “ reverse T2DM” or “diabetes be reversed” or “diabetes mellitus be reversed” or “type 2 diabetes be reversed” or “type 2 diabetes mellitus be reversed” or “T2DM be reversed”)or((“Type 1 Diabetes"or"Diabetes, Type 1"or"Diabetes Mellitus, Insulin-Dependent"or"Diabetes Mellitus, Insulin Dependent"or"Insulin-Dependent Diabetes Mellitus"or"Diabetes Mellitus, Juvenile-Onset"or"Diabetes Mellitus, Juvenile Onset"or"Juvenile-Onset Diabetes Mellitus"or"IDDM"or"Diabetes Mellitus, Type I"or"Diabetes Mellitus, Sudden-Onset"or"Diabetes Mellitus, Sudden Onset"or"Sudden-Onset Diabetes Mellitus"or"Type 1 Diabetes Mellitus"or"Diabetes Mellitus, Insulin-Dependent, 1"or"Insulin-Dependent Diabetes Mellitus 1"or"Insulin Dependent Diabetes Mellitus 1"or"Juvenile-Onset Diabetes"or"Diabetes, Juvenile-Onset"or"Juvenile Onset Diabetes"or"Diabetes, Autoimmune"or"Autoimmune Diabetes"or"Diabetes Mellitus, Brittle"or"Brittle Diabetes Mellitus"or"Diabetes Mellitus, Ketosis-Prone"or"Diabetes Mellitus, Ketosis Prone"or"Ketosis-Prone Diabetes Mellitus"or"Diabetes Mellitus, Adult-Onset"or"Adult-Onset Diabetes Mellitus"or"Diabetes Mellitus, Adult Onset"or"Diabetes Mellitus, Ketosis-Resistant"or"Diabetes Mellitus, Ketosis Resistant"or"Ketosis-Resistant Diabetes Mellitus"or"NIDDM"or"Diabetes Mellitus, Maturity-Onset"or"Diabetes Mellitus, Maturity Onset"or"Diabetes Mellitus, Non Insulin Dependent"or"Diabetes Mellitus, Non-Insulin-Dependent"or"Non-Insulin-Dependent Diabetes Mellitus"or"Diabetes Mellitus, Noninsulin Dependent"or"Diabetes Mellitus, Noninsulin-Dependent"or"Diabetes Mellitus, Slow-Onset"or"Diabetes Mellitus, Slow Onset"or"Slow-Onset Diabetes Mellitus"or"Diabetes Mellitus, Stable"or"Stable Diabetes Mellitus"or"Diabetes Mellitus, Type II"or"Maturity-Onset Diabetes"or"Diabetes, Maturity-Onset"or"Maturity Onset Diabetes"or"Maturity-Onset Diabetes Mellitus"or"Maturity Onset Diabetes Mellitus"or"MODY (Maturity-onset diabetes of the young)"or"Noninsulin-Dependent Diabetes Mellitus"or"Noninsulin Dependent Diabetes Mellitus"or"Type 2 Diabetes"or"Diabetes, Type 2"or"Type 2 Diabetes Mellitus" )and(“reversal”or“remission”or“reverse”)))

Document types：article

Pubmed

(“diabetes remission”[Title] or “diabetes mellitus remission” [Title]or “type 2 diabetes remission”[Title] or “type 2 diabetes mellitus remission”[Title] or “T2DM remission”[Title] or “remission of diabetes”[Title] or “remission of diabetes mellitus”[Title] or“remission of type 2 diabetes”[Title] or“remission of type 2 diabetes mellitus”[Title] or“reversal of diabetes”[Title] or“reversal of diabetes mellitus”[Title] or“reversal of type 2 diabetes”[Title] or“reversal of type 2 diabetes mellitus”[Title] or“reversal of T2DM”[Title] or“reverse diabetes”[Title] or“reverse diabetes mellitus”[Title] or“reverse type 2 diabetes”[Title] or“ reverse type 2 diabetes mellitus”[Title] or“ reverse T2DM”[Title] or“diabetes be reversed”[Title] or“diabetes mellitus be reversed”[Title] or“type 2 diabetes be reversed”[Title] or“type 2 diabetes mellitus be reversed”[Title] or“T2DM be reversed”[Title])or((“Type 1 Diabetes”[Title] or"Diabetes, Type 1”[Title] or"Diabetes Mellitus, Insulin-Dependent”[Title] or"Diabetes Mellitus, Insulin Dependent”[Title] or"Insulin-Dependent Diabetes Mellitus”[Title] or"Diabetes Mellitus, Juvenile-Onset”[Title] or"Diabetes Mellitus, Juvenile Onset”[Title] or"Juvenile-Onset Diabetes Mellitus”[Title] or"IDDM”[Title] or"Diabetes Mellitus, Type I”[Title] or"Diabetes Mellitus, Sudden-Onset”[Title] or"Diabetes Mellitus, Sudden Onset”[Title] or"Sudden-Onset Diabetes Mellitus”[Title] or"Type 1 Diabetes Mellitus”[Title] or"Diabetes Mellitus, Insulin-Dependent, 1”[Title] or"Insulin-Dependent Diabetes Mellitus 1”[Title] or"Insulin Dependent Diabetes Mellitus 1”[Title] or"Juvenile-Onset Diabetes”[Title] or"Diabetes, Juvenile-Onset”[Title] or"Juvenile Onset Diabetes”[Title] or"Diabetes, Autoimmune”[Title] or"Autoimmune Diabetes”[Title] or"Diabetes Mellitus, Brittle”[Title] or"Brittle Diabetes Mellitus”[Title] or"Diabetes Mellitus, Ketosis-Prone”[Title] or"Diabetes Mellitus, Ketosis Prone”[Title] or"Ketosis-Prone Diabetes Mellitus”[Title] or"Diabetes Mellitus, Adult-Onset”[Title] or"Adult-Onset Diabetes Mellitus”[Title] or"Diabetes Mellitus, Adult Onset”[Title] or"Diabetes Mellitus, Ketosis-Resistant”[Title] or"Diabetes Mellitus, Ketosis Resistant”[Title] or"Ketosis-Resistant Diabetes Mellitus”[Title] or"NIDDM”[Title] or"Diabetes Mellitus, Maturity-Onset”[Title] or"Diabetes Mellitus, Maturity Onset”[Title] or"Diabetes Mellitus, Non Insulin Dependent”[Title] or"Diabetes Mellitus, Non-Insulin-Dependent”[Title] or"Non-Insulin-Dependent Diabetes Mellitus”[Title] or"Diabetes Mellitus, Noninsulin Dependent”[Title] or"Diabetes Mellitus, Noninsulin-Dependent”[Title] or"Diabetes Mellitus, Slow-Onset”[Title] or"Diabetes Mellitus, Slow Onset”[Title] or"Slow-Onset Diabetes Mellitus”[Title] or"Diabetes Mellitus, Stable”[Title] or"Stable Diabetes Mellitus”[Title] or"Diabetes Mellitus, Type II”[Title] or"Maturity-Onset Diabetes”[Title] or"Diabetes, Maturity-Onset”[Title] or"Maturity Onset Diabetes”[Title] or"Maturity-Onset Diabetes Mellitus”[Title] or"Maturity Onset Diabetes Mellitus”[Title] or"MODY (Maturity-onset diabetes of the young)”[Title] or"Noninsulin-Dependent Diabetes Mellitus”[Title] or"Noninsulin Dependent Diabetes Mellitus”[Title] or"Type 2 Diabetes”[Title] or"Diabetes, Type 2”[Title] or"Type 2 Diabetes Mellitus" )and(“reversal”[Title] or“remission”[Title] or“reverse”[Title]))

Document types：“Adaptive Clinical Trial” or “Clinical Trial” or “Clinical Trial, Phase I - Ⅳ” or “Clinical Trial, Veterinary” or “Controlled Clinical Trial” or “Equivalence Trial” or “Multicenter Study” or “Pragmatic Clinical Trial” or “Randomized Controlled Trial” or “Randomized Controlled Trial, Veterinary” or “Case Reports” or “Clinical Study” or “Comparative Study” or “Evaluation Study” or “Observational Study” or “Observational Study, Veterinary” or “Twin Study” or “Validation Study” or “Clinical Trial Protocol” or “Dataset” or “Preprint” or “Technical Report”

Scopus

TITLE((“diabetes remission” or “diabetes mellitus remission” or “type 2 diabetes remission” or “type 2 diabetes mellitus remission” or “T2DM remission” or “remission of diabetes” or “remission of diabetes mellitus” or “remission of type 2 diabetes” or “remission of type 2 diabetes mellitus” or “reversal of diabetes” or “reversal of diabetes mellitus” or “reversal of type 2 diabetes” or “reversal of type 2 diabetes mellitus” or “reversal of T2DM” or “reverse diabetes” or “reverse diabetes mellitus” or “reverse type 2 diabetes” or “ reverse type 2 diabetes mellitus” or “ reverse T2DM” or “diabetes be reversed” or “diabetes mellitus be reversed” or “type 2 diabetes be reversed” or “type 2 diabetes mellitus be reversed” or “T2DM be reversed”)or((“Type 1 Diabetes"or"Diabetes, Type 1"or"Diabetes Mellitus, Insulin-Dependent"or"Diabetes Mellitus, Insulin Dependent"or"Insulin-Dependent Diabetes Mellitus"or"Diabetes Mellitus, Juvenile-Onset"or"Diabetes Mellitus, Juvenile Onset"or"Juvenile-Onset Diabetes Mellitus"or"IDDM"or"Diabetes Mellitus, Type I"or"Diabetes Mellitus, Sudden-Onset"or"Diabetes Mellitus, Sudden Onset"or"Sudden-Onset Diabetes Mellitus"or"Type 1 Diabetes Mellitus"or"Diabetes Mellitus, Insulin-Dependent, 1"or"Insulin-Dependent Diabetes Mellitus 1"or"Insulin Dependent Diabetes Mellitus 1"or"Juvenile-Onset Diabetes"or"Diabetes, Juvenile-Onset"or"Juvenile Onset Diabetes"or"Diabetes, Autoimmune"or"Autoimmune Diabetes"or"Diabetes Mellitus, Brittle"or"Brittle Diabetes Mellitus"or"Diabetes Mellitus, Ketosis-Prone"or"Diabetes Mellitus, Ketosis Prone"or"Ketosis-Prone Diabetes Mellitus"or"Diabetes Mellitus, Adult-Onset"or"Adult-Onset Diabetes Mellitus"or"Diabetes Mellitus, Adult Onset"or"Diabetes Mellitus, Ketosis-Resistant"or"Diabetes Mellitus, Ketosis Resistant"or"Ketosis-Resistant Diabetes Mellitus"or"NIDDM"or"Diabetes Mellitus, Maturity-Onset"or"Diabetes Mellitus, Maturity Onset"or"Diabetes Mellitus, Non Insulin Dependent"or"Diabetes Mellitus, Non-Insulin-Dependent"or"Non-Insulin-Dependent Diabetes Mellitus"or"Diabetes Mellitus, Noninsulin Dependent"or"Diabetes Mellitus, Noninsulin-Dependent"or"Diabetes Mellitus, Slow-Onset"or"Diabetes Mellitus, Slow Onset"or"Slow-Onset Diabetes Mellitus"or"Diabetes Mellitus, Stable"or"Stable Diabetes Mellitus"or"Diabetes Mellitus, Type II"or"Maturity-Onset Diabetes"or"Diabetes, Maturity-Onset"or"Maturity Onset Diabetes"or"Maturity-Onset Diabetes Mellitus"or"Maturity Onset Diabetes Mellitus"or"MODY (Maturity-onset diabetes of the young)"or"Noninsulin-Dependent Diabetes Mellitus"or"Noninsulin Dependent Diabetes Mellitus"or"Type 2 Diabetes"or"Diabetes, Type 2"or"Type 2 Diabetes Mellitus" )and(“reversal”or“remission”or“reverse”)))

Document types：article
