# Supplementary material for: Knowledge evolution and emerging emphasis on outcome standardization in diabetes remission research: a multidatabase bibliometric analysis, 1950 to January 2026
Source: Front Endocrinol (Lausanne). 2026 Oct 13;17:1943422. doi: 10.3389/fendo.2026.1943422 (PMC13711509; doi:10.3389/fendo.2026.1943422)
Supplement: Supplementary file 2 [file Supplementaryfile2.docx]

****Supplementary Table 1. Annual publication volume in the field of diabetes remission (1950–2026)****

| **Year** | **Count** | **Year** | **Count** |
| --- | --- | --- | --- |
| 1953 | 1 | 1993 | 11 |
| 1954 | 1 | 1994 | 12 |
| 1956 | 2 | 1995 | 5 |
| 1957 | 1 | 1996 | 4 |
| 1959 | 2 | 1997 | 9 |
| 1960 | 1 | 1998 | 3 |
| 1961 | 2 | 1999 | 11 |
| 1962 | 3 | 2000 | 10 |
| 1963 | 1 | 2001 | 15 |
| 1964 | 3 | 2002 | 11 |
| 1965 | 2 | 2003 | 10 |
| 1967 | 2 | 2004 | 14 |
| 1969 | 1 | 2005 | 14 |
| 1971 | 2 | 2006 | 20 |
| 1972 | 1 | 2007 | 11 |
| 1973 | 1 | 2008 | 19 |
| 1974 | 3 | 2009 | 23 |
| 1976 | 2 | 2010 | 20 |
| 1977 | 1 | 2011 | 39 |
| 1978 | 5 | 2012 | 56 |
| 1979 | 4 | 2013 | 37 |
| 1980 | 3 | 2014 | 47 |
| 1981 | 6 | 2015 | 71 |
| 1982 | 8 | 2016 | 63 |
| 1983 | 1 | 2017 | 73 |
| 1984 | 6 | 2018 | 71 |
| 1985 | 2 | 2019 | 83 |
| 1986 | 7 | 2020 | 100 |
| 1987 | 9 | 2021 | 111 |
| 1988 | 10 | 2022 | 98 |
| 1989 | 5 | 2023 | 101 |
| 1990 | 10 | 2024 | 121 |
| 1991 | 9 | 2025 | 127 |
| 1992 | 6 | 2026 | 11 |

****Supplementary Table 2. Top 10 countries/regions with the highest number of publications in the field of diabetes remission****

| Country/Region | Count | Centrality | Citations | Total Link Strength |
| --- | --- | --- | --- | --- |
| USA | 365 | 0.15 | 21,080 | 186 |
| China | 176 | 0.00 | 4,045 | 50 |
| United Kingdom | 132 | 0.04 | 8,142 | 114 |
| Italy | 63 | 0.03 | 3,735 | 82 |
| Canada | 61 | 0.08 | 5,168 | 55 |
| Germany | 58 | 0.09 | 2,269 | 87 |
| Japan | 47 | 0.02 | 1,624 | 29 |
| India | 42 | 0.00 | 572 | 14 |
| Spain | 35 | 0.00 | 1,030 | 28 |
| Denmark | 29 | 0.01 | 2,450 | 58 |
| France | 26 | 0.17 | 1,119 | 37 |
| Australia | 25 | 0.00 | 1,388 | 27 |
| Sweden | 22 | 0.03 | 2,917 | 67 |
| Brazil | 11 | 0.00 | 455 | 20 |

****Supplementary Table 3. Top 15 institutions with the highest number of publications in the field of diabetes remission****

| Institution | Country | Count |
| --- | --- | --- |
| Newcastle University | UK | 58 |
| University of Glasgow | UK | 46 |
| Harvard University | USA | 33 |
| University of Florida | USA | 28 |
| University of Washington | USA | 22 |
| Karolinska Institutet | Sweden | 18 |
| CIBER | Spain | 17 |
| Central South University | China | 17 |
| University College Dublin | Ireland | 16 |
| Massachusetts General Hospital | USA | 13 |
| University of Copenhagen | Denmark | 13 |
| McMaster University | Canada | 12 |
| Pennington Biomedical Research Center | USA | 12 |
| Mayo Clinic | USA | 11 |
| Poznan University of Medical Sciences | Poland | 11 |

****Supplementary Table 4. Keywords ranked by frequency****

| Keyword | Freq |
| --- | --- |
| diabetes mellitus | 906 |
| human | 812 |
| male | 713 |
| female | 659 |
| remission | 617 |
| non insulin dependent diabetes mellitus | 505 |
| insulin | 184 |
| controlled study | 165 |
| adult | 118 |
| nonhuman | 74 |

**Supplementary Table 5. Bibliometric evidence supporting the temporal interpretation of diabetes remission research**

| Descriptive period | Documented co-citation pattern | Representative burst reference | Publication year | Burst period | Burst strength | Interpretive relevance |
| --- | --- | --- | --- | --- | --- | --- |
| Early accumulation (≤2000) | Earlier knowledge-base clusters included “cure,” “mechanism,” and “mediating remission.” | No representative top citation burst began within this descriptive period in the supplied CiteSpace burst output. | — | — | — | The early literature was interpreted primarily as establishing whether remission/reversal was possible and exploring mechanisms. This interpretation is supported mainly by the earlier co-citation structure rather than by a strong citation burst beginning before 2001. |
| Transitional growth (2001–2010) | The knowledge base moved from earlier cure/mechanism themes toward metabolic and physiological explanations. | Schölin A et al., Journal of Internal Medicine | 1999 | 2001–2004 | 1.27 | An early burst extending into the transitional period illustrates continued attention to clinical remission-related questions at the start of the 2000s. |
| Transitional growth (2001–2010) | Intermediate themes increasingly reflected metabolic remodeling and physiology. | Dixon JB et al., JAMA — randomized trial of adjustable gastric banding | 2008 | 2008–2012 | 3.13 | The strongest burst in the analysis supports the growing influence of intervention-based evidence on diabetes remission during the transition toward expansion. |
| Transitional growth (2001–2010) | Intermediate themes increasingly reflected metabolic remodeling and physiology. | Adams TD et al., New England Journal of Medicine — long-term mortality after gastric bypass | 2007 | 2009–2012 | 2.21 | The burst supports increasing attention to longer-term outcomes of metabolic surgery. |
| Transitional growth (2001–2010) | The knowledge base began to include explicit discussion of how diabetes cure/remission should be defined. | Buse JB et al., Diabetes Care — “How do we define cure of diabetes?” | 2009 | 2011–2013 | 1.11 | Although the burst began after 2010, the publication itself arose from the transitional-period literature and provides an early bridge toward later emphasis on remission terminology and standardization. |
| Expansion (2011–2025) | Recent co-citation clusters included “defining remission,” “duration,” and “long-term follow-up.” | Schauer PR et al., New England Journal of Medicine — 5-year outcomes of bariatric surgery versus intensive medical therapy | 2017 | 2017–2021 | 2.10 | Supports sustained interest in comparative effectiveness and longer-term remission outcomes during the expansion period. |
| Expansion (2011–2025) | Recent clusters increasingly emphasized remission, durability, and long-term follow-up. | Lean MEJ et al., Lancet — DiRECT trial | 2018 | 2020–2023 | 1.56 | Supports the expansion of non-surgical weight-management approaches and the use of remission as an explicit clinical outcome. |
| Expansion (2011–2025) | Recent clusters included “defining remission.” | Nagi D et al., British Journal of Diabetes — UK position statement on type 2 diabetes remission | 2019 | 2020–2021 | 1.53 | Provides bibliometric evidence for increasing attention to terminology, clinical interpretation, and standardization of remission. |
| Expansion (2011–2025) | Recent clusters emphasized “duration” and “long-term follow-up.” | Lean MEJ et al., Lancet Diabetes & Endocrinology — 2-year DiRECT follow-up | 2019 | 2021–2023 | 1.40 | Supports a shift from demonstrating initial remission toward evaluating its durability and maintenance. |
| Expansion (2011–2025) | Recent clusters emphasized “long-term follow-up.” | Mingrone G et al., Lancet — 10-year follow-up of metabolic surgery | 2021 | 2021–2026 | 1.76 | Extends the evidence base toward long-term durability of diabetes remission after metabolic surgery. |
| Expansion (2011–2025) | Recent clusters included “defining remission.” | Riddle MC et al., Diabetes Care — Consensus Report: Definition and Interpretation of Remission in Type 2 Diabetes | 2021 | 2021–2026 | 1.76 | Provides the most direct bibliometric support for the interpretation that definition and standardization of remission became increasingly prominent. |
| Expansion (2011–2025) | The recent knowledge base broadened beyond surgery and lifestyle interventions. | Frías JP et al., New England Journal of Medicine — tirzepatide versus semaglutide | 2021 | 2023–2026 | 1.65 | Indicates broadening of the contemporary knowledge base toward newer glucose- and weight-lowering therapies; it is not interpreted as a remission-definition study. |

****Supplementary Table 6**. Comparison of the present study with bibliometric studies published in 2023 and 2025**

| Comparison item | Yang et al., 2023 | Liu et al., 2025 | Present study |
| --- | --- | --- | --- |
| Research scope | Diabetes remission, including type 1- and type 2-related research | Type 2 diabetes remission | Diabetes remission, including type 1- and type 2-related research |
| Time span | 2000-April 2023 | 2002-2022 | 1950-January 2026 |
| Database | Web of Science | Web of Science | WoSCC, Scopus, and PubMed |
| Document type and sample size | 970 English-language articles | 1,903 articles and 351 reviews; 2,254 publications in total | 1,554 original articles |
| Main tools | CiteSpace and VOSviewer | Bibliometrix and CiteSpace | VOSviewer, CiteSpace, Bibliometrix, and SCImago Graphica |
| Leading country | United States ,342 publications; followed by China and the United Kingdom | United States, 476; China, 347; United Kingdom, 180 | United States, 365; China, 176; United Kingdom, 132 |
| Leading institution | Newcastle University, 36 publications | University of Copenhagen, 116 publications | Newcastle University, 58 publications; University of Glasgow, 46 publications |
| Leading author | Roy Taylor, 35 publications | Lee, 48 publications; Roy Taylor, 37 publications | Roy Taylor, 37 publications |
| Leading journal | Obesity Surgery, 81 publications | Obesity Surgery, 296 publications | Obesity Surgery, 116 publications |
| Core themes | Metabolic surgery and lifestyle intervention; an apparent shift from basic experiments to clinical research | Bariatric surgery, weight loss, and remission; continuing attention to GLP-1 receptor agonists and low-energy diets | Confirmation of these themes, with additional characterization of early case and transplantation evidence, mechanistic development, remission definitions, durability, and long-term follow-up |
| Main contribution | An initial overall knowledge map of the field | Thematic evolution and burst terms focused on type 2 diabetes remission | Multidatabase coverage, a longer time span, and cross-interpretation of several temporal signals, supporting an evolutionary framework from documentation of remission to increasing attention to outcome standardization |
